# Dexamethasone-Mediated Activation of Fibronectin Matrix Assembly Reduces Dispersal of Primary Human Glioblastoma Cells

**DOI:** 10.1371/journal.pone.0135951

**Published:** 2015-08-18

**Authors:** Stephen Shannon, Connan Vaca, Dongxuan Jia, Ildiko Entersz, Andrew Schaer, Jonathan Carcione, Michael Weaver, Yoav Avidar, Ryan Pettit, Mohan Nair, Atif Khan, Ramsey A. Foty

**Affiliations:** 1 Department of Surgery, Rutgers-Robert Wood Johnson Medical School, New Brunswick, NJ, United States of America; 2 Department of Radiation Oncology, Cancer Institute of New Jersey, Rutgers-Robert Wood Johnson Medical School, New Brunswick, NJ, United States of America; Thomas Jefferson University, UNITED STATES

## Abstract

Despite resection and adjuvant therapy, the 5-year survival for patients with Glioblastoma multiforme (GBM) is less than 10%. This poor outcome is largely attributed to rapid tumor growth and early dispersal of cells, factors that contribute to a high recurrence rate and poor prognosis. An understanding of the cellular and molecular machinery that drive growth and dispersal is essential if we are to impact long-term survival. Our previous studies utilizing a series of immortalized GBM cell lines established a functional causation between activation of fibronectin matrix assembly (FNMA), increased tumor cohesion, and decreased dispersal. Activation of FNMA was accomplished by treatment with Dexamethasone (Dex), a drug routinely used to treat brain tumor related edema. Here, we utilize a broad range of qualitative and quantitative assays and the use of a human GBM tissue microarray and freshly-isolated primary human GBM cells grown both as conventional 2D cultures and as 3D spheroids to explore the role of Dex and FNMA in modulating various parameters that can significantly influence tumor cell dispersal. We show that the expression and processing of fibronectin in a human GBM tissue-microarray is variable, with 90% of tumors displaying some abnormality or lack in capacity to secrete fibronectin or assemble it into a matrix. We also show that low-passage primary GBM cells vary in their capacity for FNMA and that Dex treatment reactivates this process. Activation of FNMA effectively “glues” cells together and prevents cells from detaching from the primary mass. Dex treatment also significantly increases the strength of cell-ECM adhesion and decreases motility. The combination of increased cohesion and decreased motility discourages in vitro and ex vivo dispersal. By increasing cell-cell cohesion, Dex also decreases growth rate of 3D spheroids. These effects could all be reversed by an inhibitor of FNMA and by the glucocorticoid receptor antagonist, RU-486. Our results describe a new role for Dex as a suppressor of GBM dispersal and growth.

## Introduction

Glioblastoma is an aggressive disease with a high mortality rate. Despite advances in surgery, radiotherapy and adjuvant chemotherapy, median survival of patients diagnosed with GBM is less than 10% at five years [1]. These dismal statistics largely reflect an inability to provide effective local treatment as GBM cell dispersal into the brain parenchyma occurs early in the disease process [2]. As a consequence, tumors typically recur close to the operative site. Re-operation for recurrence yields little survival advantage [3] due to the continued and ongoing spread of these cells from the recurrent mass. Patients ultimately succumb to GBM due to two major factors- continued dispersal, and rapid growth of the recurrence. Continued dispersal renders targeted therapy largely ineffective, whereas rapid growth ultimately gives rise to increased intracranial pressure due to mass effect. Therapeutic strategies that target molecular and cellular processes mediating dispersal and growth are needed if post-operative disease-free and overall survival in these patients is to be improved. Various factors can influence dispersal and growth, including the strength of cell-cell cohesion [4], cell-ECM adhesion [5], cell motility [6], and to some extent, the stiffness of individual tumor cells [7] and of the ECM [8]. A decrease in the ability of cells to detach from a primary mass (mediated by increased cell-cell cohesion), coupled with an effective decrease in cell motility (mediated by increased cell-ECM attachment) could, in principle, reduce dispersal. Moreover, if the stiffness of dispersing cells could be manipulated to render them less able to physically migrate through their microenvironment, this could also reduce their ability to disperse. A drug that can cross the blood-brain barrier and influence cell dispersal and tumor growth would be an ideal therapeutic candidate [4, 9, 10].

Both α5β1 integrin and fibronectin are upregulated in Glioblastoma. α5β1 integrin is typically expressed in a perinecrotic or perivascular pattern [11] and its expression has been shown to both facilitate [12] and inhibit [13] glioma cell migration. This dichotomy in function may be explained by inherent differences between cell types or by differences in the composition of the ECM [12, 13]. For example, α5-neutralizing antibodies inhibited invasion of D37MG [12], increased invasion of U138MG [13], and had no effect in U251.3MG [14]. In the *in vitro* experiments in which α5 neutralizing antibodies reduced migration, the cells were plated onto purified fibronectin. Whereas, in the setting where α5 glioma invasion was enhanced [13], glioma cells were plated onto Matrigel, which contains not only fibronectin, but numerous other ECM proteins. Accordingly, other integrins expressed by glioma cells may also influence α5-mediated. Thus, tumors derived from different cell lines can have vastly different capacity for cell migration and antagonizing α5 integrin may not consistently influence migration in one direction or another. More recent studies using global integrin phenotyping identified α3β1 as the predominant integrin mediating the dispersal of glioma cells and that treating U251 glioma cells with neutralizing antibodies to α3β1 prior to injection into nude mice inhibited invasion into brain tissue [15]. These data suggest that α5β1-fibronectin interaction may not necessarily be of fundamental importance to glial cell migration. However, previous studies from our group have shown that activating α5 integrin function in cell lines lacking capacity for fibronectin matrix assembly (FNMA) consistently reduced tumor cell dispersal and migration. Dexamethasone (Dex), a drug routinely used to treat brain tumor-related edema, is highly effective in activating FNMA in human fibrosarcoma (HT-1080) [9], rat prostate cancer cells [10], and various GBM cell lines [4]. Thus, a strategy of pharmacologically activating fibronectin matrix assembly consistently gave rise to reduced dispersal irrespective of cell type. Other studies from our group demonstrated that irrespective of the strength of cell-ECM adhesion, a modest increase in cell-cell cohesion was sufficient to significantly reduce dispersal [5].

In 3D spheroid cultures, an increase in FNMA would effectively cross-link cells together to increase bulk-cohesion of the spheroid [16]. Previous studies using U87-MG, a GBM cell line developed nearly 50 years ago, showed that Dex-treatment resulted in markedly increased cohesion and reduced dispersal velocity (DV). Of note, is that the pattern of dispersal was affected by Dex treatment. The advancing edge of untreated aggregates dispersed as single cells, whereas the leading edge of Dex-treated aggregates advanced as a sheet. Cells at the advancing front were tightly adherent to one another, suggesting that the Dex-mediated decrease in DV arose as a consequence of increased cell-cell cohesion [4]. These previous studies implicated FNMA as a potential mediator of GBM dispersal but were limited by the fact that they were based entirely on cells that may no longer be comparable to GBM cells in an actual tumor. Little is known as to whether reduced capacity for FNMA exists within relevant clinical samples, or whether freshly-excised primary GBM cells would respond to Dex treatment in a manner similar to that observed for immortalized GBM cells. In this study, we explored FN expression patterns in a human GBM tumor microarray and in low-passage human primary GBM cells. We asked whether these lines differed in properties that could influence their capacity to disperse. Such properties include strength of intercellular cohesion, strength of cell adhesion to the ECM, and cell motility. We also assessed the effects of Dex treatment on cell-cell cohesion, cell-substratum adhesion, cell motility, in vitro and ex vivo dispersal, and on growth rate of 3D spheroids.

## Materials and Methods

### Assessment of fibronectin expression patterns in a Glioblastoma tumor microarray

A serial GBM tissue array consisting of 40 cases/80 cores with a pathological diagnosis of GBM (US Biomax, GL806) was probed with an anti-fibronectin antibody, followed by a FITC-conjugated secondary antibody. The microarray consists of 3 cores GBM Grade 3–4, 30 cores GBM Grade 4, and 5 cores normal cerebrum (in duplicate). According to the manufacturer, every 10th section of the tissue array is stained with H&E and reviewed by two board certified pathologists to confirm that the pathology diagnosis is current and matched to the adjacent serial sections. Low-magnification images were captured for each 0.6 mm GBM core using a Nikon Eclipse inverted fluorescence microscope attached to an QImaging EXi Blue digital camera and an Apple iMac computer running iVision-Mac Image analysis software. Images representing the predominant pattern of fibronectin expression in each section were collected. The corrected fluorescence intensity for all images was measured in ImageJ and analyzed by k-means clustering in Statistica. Cores from each cluster were re-examined and images were captured at higher magnifications to better illustrate FN expression patterns.

### Primary GBM cells: generation, characterization, and culture

Primary GBM tumor samples were obtained with approval of the Rutgers-Robert Wood Johnson Medical School Institutional Review Board under protocol #CINJ 001208. Samples were anonymized and the IRB waived the need for written informed consent. The lines used in this study have previously been published in [17]. Samples were examined by a neuropathologist and stained for GFAP to confirm their designation as GBM. Primary cells from part of the tumor were isolated by cutting the tumor into small fragments followed by dissociation in 0.05% trypsin-0.1mM EDTA (TE). Single cells were then plated into serum-containing medium. In the presence of serum, cells attached to the tissue culture plate and were positive for GFAP, indicating glial origin. GFAP has also been demonstrated to be a reliable marker in GBM [18]. These cells were expanded and used exclusively at 3^rd^– 6^th^ passage. Cells were maintained in EMEM/10% FCS/antibiotic/antimycotic and passaged by dissociation with TE using standard protocols. Normal human astrocytes (NHA) were purchased from Life Technologies (Carlsbad, CA) and maintained in astrocyte medium (88% DMEM, 1.0% N-2 Supplement, 10% FBS, 1% AA, and 2 μg/100mL EGF). For routine propagation, tissue culture dishes were coated with 200 μL of Geltrex LDEV-free Reduced Growth Factor Basement Membrane Matrix (Life Technologies, Carlsbad, CA) per 1.0 cm^2^ of surface area and washed with phosphate-buffered saline (PBS) prior to cell seeding. Cells were passaged using StemPro Accutase Cell Dissociation Reagent (Life technologies, Carlsbad, CA).

### Dexamethasone, FUD, and RU-486 treatment

A typical therapeutic oral dose of Dex is between 4 and 16 mg/day. The predicted concentration of Dex in the brain 6 hours after a 0.5 mg oral dose of Dex peaks at 5x10^-3^ μg/ml [19]. This is equivalent to a 1.3x10^-8^ M in vitro concentration. Therefore, a 4 mg dose is equivalent to an in vitro concentration of 1x10^-7^ M. Cells were plated at 70% confluence and incubated for 24 hours, whereupon Dex (Sigma, MO, USA) was added from a 1x10^-3^ M stock to a final concentration of 1x10^-7^ M. The 49-residue functional upstream domain (FUD) of Streptococcus pyrogenes F1 adhesin has previously been shown to prevent assembly of a FN matrix [20, 21]. A recombinant FUD (pUR-4) [22] was obtained from Dr. Jane Sottile through a UBMTA between the University of Rochester and Rutgers-RWJMS. FUD was added to tissue culture medium at a final concentration of 1 μg/ml. Cells were incubated with Dex or Dex+FUD overnight, prior to fixation. RU-486 (Abcam, Cambridge, MA) was added to culture medium at a final concentration of 1 mM weight/volume in DMSO.

### Generation of 3D spheroids

Cells were removed from near-confluent plates with TE, washed, and suspended at a concentration of 2.5 x10^6^ cells/ml in complete medium supplemented with 2 mM CaCl_2_. Ten-microliters were deposited on the underside of a 10-cm tissue culture dish lid. The lid was then inverted over 10 ml of PBS for hydration. Hanging drops were incubated under tissue culture conditions for 48 h, allowing the cells to coalesce at the base of the droplets and to form multilayer aggregates. These were then transferred to agarose-coated plates and incubated for several more days until aggregates formed spheres. Tissue surface tension measurements were performed using spheres that had been incubated on agarose for 2–4 days.

### Measurement of aggregate cohesion by tissue surface tensiometry (TST)

The method has been explained in detail in [23–26]. TST was developed to generate rigorous measurements of intercellular binding energy in 3D tissue-like spheroids under physiological conditions. Briefly, mutually cohesive cells, if prevented from adhering to a substrate, will spontaneously assemble into clusters. Over time, these clusters will “round up” to form spheres, a behavior typical of liquids. They do so to minimize their surface area to volume ratio and thus, adhesive free-energy. TST employs a custom-built instrument to compress spherical cellular aggregates between parallel plates to which they cannot adhere. Measurements of aggregate geometry and resistance to the applied force are then used to establish tissue liquidity and to calculate aggregate surface tension. Sixteen to twenty aggregates from each GBM line were used to generate an average surface tension and attendant standard deviations and standard errors.

### Validation of surface tension measurements

The calculated surface tension of a liquid aggregate, when subjected to two successive compressions (σ_1_ and σ_2_), the second greater than the first, will remain constant. In such aggregates the ratio of σ_2_/σ_1_ will approach 1 and will be less than the ratio of the force applied at each successive compression (F_2_/F_1_). In contrast, the calculated surface tension of an elastic aggregate will obey Hooke's law and increase proportionately to the applied force. For elastic aggregates the ratio of σ_2_/σ_1_ will not be equal to 1 but will instead approach the ratio of F_2_/F_1_. The surface tension of liquid aggregates will also be independent of aggregate size. Only measurements in which surface tension is independent of the applied force and size were used to calculate average σ for each cell line.

### Assessment of FNMA by GBM cells

FNMA was assessed by deoxycholic acid (DOC) differential solubilization [9, 16]. For 2D cultures, cells were plated in fibronectin-containing tissue culture medium with or without 10^−7^ M Dex and incubated overnight. Near-confluent monolayers were lysed in DOC. For 3D cultures, cells were treated as described above and spheroids were generated by the hanging drop method. Fifity spheroids were collected and pooled then lysed in DOC by sonication. For both 2D and 3D cultures, 5 μg of protein were separated by SDS-PAGE using 4% stack/5% separating gels under reducing conditions. Gels were blotted to PVDF, and probed with an anti-FN antibody (ab6584, Abcam, UK) and an HRP-conjugated secondary antibody. Under reducing conditions, fibronectin resolve as a 220-kDa band. In 2D cultures, FNMA was assessed by immunofluorescence using the same primary antibody and a FITC-conjugated secondary antibody. Cells were plated at 90% confluence and incubated overnight whereupon they were washed in PBS and fixed in 4% paraformaldehyde (PFA)/0.2% Triton-X100 for 30 minutes at RT. They were then incubated in anti Fn antibody for 1 hour at RT, followed by an Alexa-Fluor-488-conjugated secondary antibody for 30 minutes at RT, with 3 PBS washes in between. DAPI was added prior to mounting. Cells were imaged by epifluorescence microscopy. For 3D cultures, FNMA was assessed by generating spheroids in the presence of 50 μg/ml Rhodamine-FN (Cytoskeleton, Inc.) for 48 hours, whereupon aggregates were fixed in 4% PFA. Aggregates were imaged using an upright Zeiss AxioImager Z1 spinning Disc confocal microscope attached to a Photometrics Evolve 512 EMCCD camera and Metamorph Premier imaging software.

### Assessment of actin organization

For 2D culture, cells were sparsely plated onto coverslips in 24-well dishes and incubated overnight, whereupon they were fixed and permeabilized in 4% PFA/0.2% Triton X-100. Cells were then immersed in 6.6 nM Rhodamine-Phalloidin in PBS for 30 minutes and counterstained with DAPI. To detect actin during dispersal from 3D spheroids, aggregates ranging in size from 50–100 μM were plated into 24 well dishes and allowed to disperse overnight, whereupon they were fixed, permeabilized, and stained as described above.

### Measurement of cell size

Fifity thousand cells were plated either in the absence or presence of Dex into wells of a 6-well plate and incubated overnight in standard tissue culture medium, whereupon they were fixed and permeabilized in 4% PFA and 0.5% TritionX-100. Cells were then stained with rhodamine-phalloidin and counterstained with DAPI. Cell size was measured by capturing images of 45 cells per group and analyzing each image using ImageJ. Nuclei served to differentiate between single cells and groups of cells. Only single cells were used to calculate average area. Student’s t-test was used to detect statistical significance between means.

### Assessment of α5β1 integrin expression

Protein lysates were prepared as previously described [10]. Twenty-five μg of protein were separated on a 7% SDS-PAGE gel under non-denaturing conditions and blotted to PVDF membranes using standard protocols. Following blocking, blots were incubated in 10 μg/ml rabbit polyclonal antibody against α5 integrin (AB1928, Chemicon, Temecula, CA) followed by an appropriate secondary antibody conjugated to horseradish peroxidase. Blots were developed using enhanced chemiluminescence and exposed to X-ray film.

### Assessment of fibronectin secretion

GBM cells were plated at equal densities in wells of a six-well dish in 2 mls of tissue culture medium (TCM) containing fetal calf serum (FCS) depleted of FN. FCS was depleted of FN by incubation with collagen-sepharose 4B beads (GE Healthcare Biosciences AB, Sweden). After 24 hours, 100 μl of tissue culture medium from each line was mixed with 100 μl (an excess) of sepharose 4B-collagen beads and rotated for 30 minutes at room temperature. Beads were washed 5 times in ice-cold PBS then boiled in SDS sample buffer containing 5% β-mercaptoethanol (BME). FN protein was separated on a 5% SDS-PAGE gel. Immunoblot analysis was then used to detect FN.

### RT-qPCR for α5 integrin

Total RNA from each cell line was isolated using the RNeasy Mini Kit (Qiagen, Valencia, CA) following the manufacturer’s protocol. The concentration and purity of RNA obtained from each cell line was determined using an Agilent Bioanalyzer. cDNA synthesis was performed using the Superscript First-Strand Synthesis System for RT-PCR (Invitrogen, Catalog #11904–018). One μg of total RNA was DNAse I treated prior to cDNA synthesis using random hexamers and Superscript II Reverse Transcriptase (SSIIRT). The reverse transcription reaction was later terminated by heating the sample at 70°C for 15 minutes followed by Rnase H treatment for 20 min. at 37°C. RT-qPCR was performed using the TaqMan Gene Expression Assay System (Applied Biosystems/Life Technologies, NY). Primers for α5-integrin and β-2 microglobulin and the TaqMan gene expression assay master mix were from Life Technologies. 100 ng of cDNA was used as template. RT-qPCR reactions were performed in a 96 well plate on a Bio-Rad CFX Connect Real Time System (Bio-Rad, Hercules, CA). The thermocycling program involved an initial 10 minute denaturation step at 95°C, followed by 40 cycles of annealing at 95°C for 30 seconds and an extension/synthesis at 60°C for one minute. β-2 microglobulin was utilized as an internal control for comparative analysis using the 2^-ΔΔCT^ method.

### Measurement of attachment to substrate

The strength of substrate attachment was measured by subjecting adhering cells to flow-induced shear stress. GBM cells were plated at a concentration of 5x10^4^ cells/ml onto 6-well Polyethylene terephthalate (PET) cell culture inserts (Franklin Lakes, NJ). The inserts have a diameter of 2.4 cm with 1.6 million 0.45 μm pores/cm^2^. Cells were allowed to attach to the inserts for 2 hours and were then inverted into complete medium and incubated overnight until they became fully attached. A similar number of cells were seeded onto inserts and incubated overnight as a no-flow growth rate control. Seeded inserts were then loaded into custom-designed flow chambers and subjected to 30 dynes/cm of shear stress for 3 hours. The inserts were removed, washed in PBS, and immersed in Syto 16 green fluorescent nucleic acid stain (Life Technologies, Carlsbad, CA). Nine low magnification fields were captured and nuclei counted in ImageJ. The average number of attached cells were then expressed as a percentage of the no-flow controls.

### Measurement of cell motility

Motility was measured using a phagokinetic assay [27]. The method utilizes 1 μM diameter non-cytotoxic fluorescent polystyrene microspheres. Tissue culture dishes were pre-coated with poly-D-lysine, whereupon beads adjusted to 0.018% v/v in PBS were adhered to the bottom of the dish. Cells were then added to the dish in complete tissue culture medium at a cell/area density of 4 cells/mm^2^. As cells move, they phagocytose the beads, creating tracks of non-fluorescent substrate. Cells become highly fluorescent due to ingestion of the beads. Cell area can therefore be measured. Cleared tracts were quantified in ImageJ by hand-tracing each non-fluorescent track. Subtracting the cell area from the cleared area normalizes the track data for any differences in cell size. Tracks generated by 20 cells were quantified for each cell line to generate an average cleared area with attendant standard deviation and standard error.

### Measurement of Dispersal Velocity

Aggregates of GBM-1-4 ranging in size from 50–100 μm were each plated into wells of 12-well tissue culture plates containing 2 mls of pre-warmed CO_2_-independent medium. Plates were incubated for 30 minutes to allow aggregates to adhere, whereupon images of each aggregate were captured every hour for 8 hours. The diameters measured were for cohorts of cells. Images were analyzed in ImageJ by measuring change in aggregate diameter over time. Dispersal velocity was determined by linear regression analysis. Only slopes with an R^2^ value of 0.95 and greater were used to calculate DV. Data was normalized with initial aggregate diameter. Twelve aggregates were used to generate an average DV.

### Assessment of *ex vivo* dispersal

Alvetex scaffolds (Reinnervate, Durham, UK) used for the *ex vivo* dispersal assay are highly porous, 200 μm thick, cross-linked polystyrene scaffolds with void diameters of 40 μm and tunnel diameters of 8–13 μm. Scaffolds were prepared by submersion in ethanol followed by rehydration with PBS and immersion in tissue culture medium at 37°C, 5.0% CO_2_ and 95% humidity for 30 minutes. Scaffolds were seeded with 1x10^6^ NHA cells in 100 μL of medium. The NHA-seeded scaffolds were kept suspended in an empty 12-well dish and incubated for 60 minutes to allow NHA cells to adhere. Wells were then filled with 4 mL medium and incubated for 48 hours to allow cells to incorporate throughout the scaffold. After 48 hours, GBM cells that had been transfected with BacMam 2.0 GFP_T_ (Life Technologies, Long Island, NY) were used to generate spheroids. These were allowed to adhere to the scaffold in a small volume of medium. The scaffolds were placed in wells of 12-well dishes containing a 1:1 mixture of NHA and GBM medium. Serum-free GBM medium was then added to the scaffold inserts. Scaffolds were incubated for 48 hours to allow time for tumor cells to infiltrate and disperse. To image dispersed cells, each scaffold was washed with PBS then cut out of its insert and mounted on a slide with 50 μL FluorSave reagent and a coverslip. A Yokogawa CSU-X1 spinning disk confocal microscope with MetaMorph software was used to capture images at successive focal planes taken at 1μm intervals to generate a Z- stack. Differential Interference Contrast (DIC) microscopy was used to identify the z = 0 starting point for each Z-stack. Each Z-stack was analyzed using ImageJ and the Z-axis position of each cell within the tissue-scaffold was scored. Within any given scaffold the mean average Z-axis cell position from 5–6 z-stacks was calculated and recorded.

### Measurement of 2D and 3D growth rates of GBM cells

Cell growth rate was measured for both conventional 2D and 3D spheroid cultures. For 2D, cells were plated at a concentration of 5x10^4^ cells/ml in wells of a 6-well dish in complete medium. Total and live cell counts were performed every day for 4 days using a BioRad TC10 automated cell counter. For 3D spheroids, aggregates were generated using the hanging drop method. Single aggregates were plated onto wells of an agarose-coated 6-well dish. Agarose prevented aggregates from adhering to the bottom of the dish. The diameter of each aggregate was measured once a day for nine days. Growth rate was determined by plotting either cell number (2D) or aggregate area (3D) as a function of time. Regression analysis was performed to calculate growth rates.

## Results

### A human GBM tissue microarray reveals 4-distinct fibronectin (FN) expression patterns

**Fig 1:**
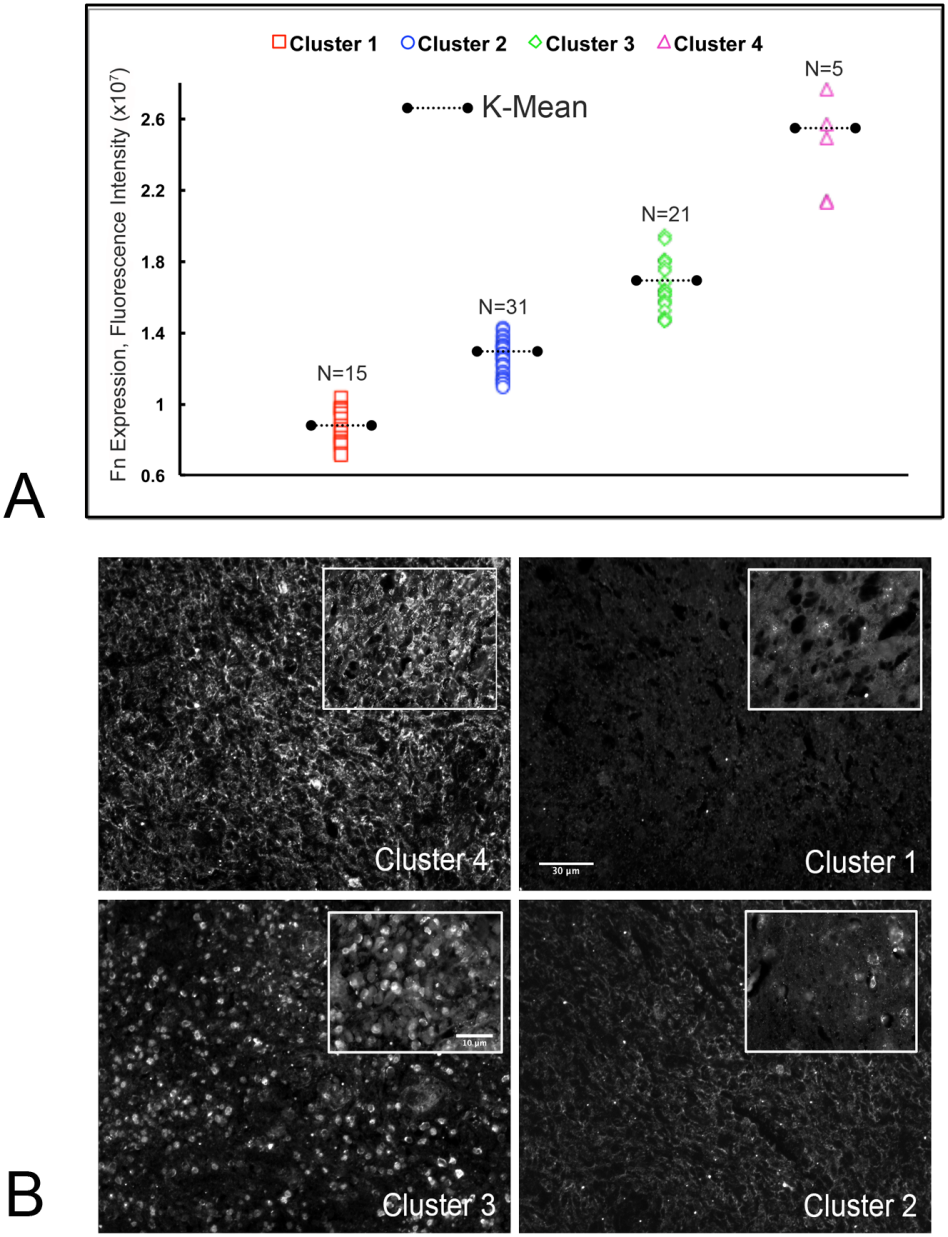
**(A)** *k*-means cluster analysis of FN expression in a 40-sample, 80-core, human GBM tissue microarray. *k*-means clustering partitions a given data set into clusters by grouping each n, into the cluster with the nearest mean, serving as a prototype of the cluster. This results in a partitioning of the data space into Voronoi cells. In this case, cluster analysis was performed for 3 and 4 Voronoi cells (3*k* and 4*k*). A *k* of 4 provided the maximum difference in center means (ANOVA, p<0.0001). **(B)** Representative immunofluorescence images of the predominant patterns of FN expression in a human GBM microarray; high FN with FNMA (Cluster 4), high intracellular FN with no FNMA (Cluster 3), low FN with reduced FNMA (Cluster 2), and low to absent FN with no FNMA (Cluster 1).

Large-scale genomic [28] and proteomic [29] studies have revealed intratumoral heterogeneity in GBM. We examined a GBM tissue array consisting of 40 cases/80 cores (US Biomax, GL806) to determine whether differences in FN expression patterns may also exist. The array was probed with an anti-fibronectin antibody, followed by a FITC-conjugated secondary antibody. Low-magnification images were captured for each core. The corrected fluorescence intensity for all images was measured and the data were analyzed by performing a k-means cluster analysis. Fig 1A shows the existence of 4 distinct clusters of corrected mean fluorescence intensity. An ANOVA revealed significant differences in mean fluorescence between the 4 groups (p<0.0001). The means for each cluster were then used to identify and characterize corresponding images (Fig 1B). The following characteristics were identified for the respective clusters: Cluster 4) FN is arranged in dense fibrils (N = 5), Cluster 3) Intracellular FN is evident without FNMA (N = 21), Cluster 2) FN forms a less extensive matrix (N = 31), C) and Cluster 1), very low to absent FN expression and/or FNMA is evident (N = 15). These data indicate that clinical samples of GBM distinctly cluster into 4 subtypes, each displaying different capacity for FN expression and organization.

### Capacity for FNMA is low in primary human GBM cells and can be activated by Dex treatment

**Fig 2:**
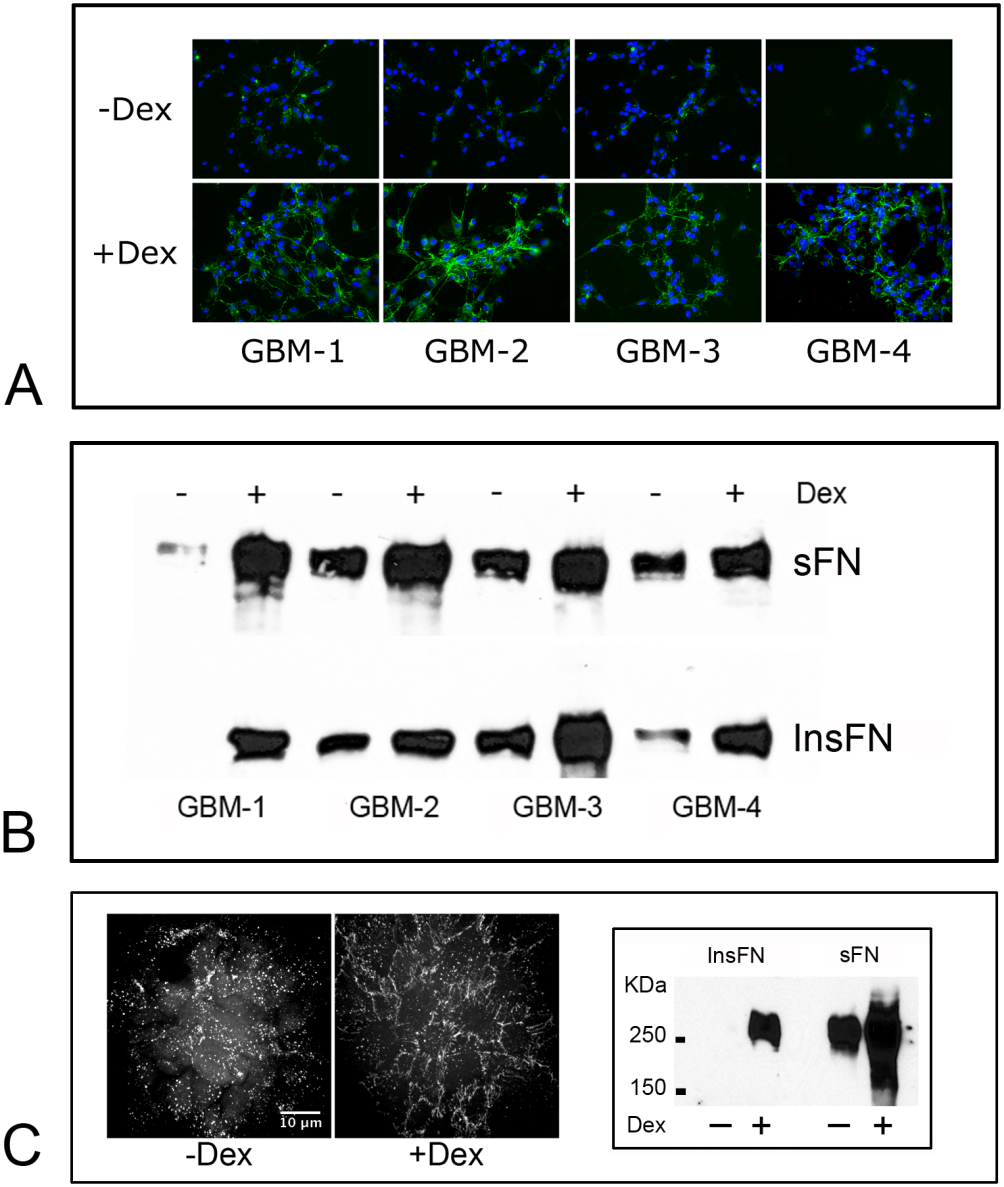
**(A)** Dex treatment activates FNMA in GBM cells. Immunofluorescence was used to compare FNMA in untreated and Dex treated GBM cells. Dex was added to a final concentration of 1x10^−7^M. Cells were treated for 24 hours prior to fixation. Dex treatment significantly increased FNMA in all lines. **(B)** Dex-mediated upregulation of FNMA was confirmed by immunoblot analysis. Lysates of untreated and Dex-treated cells were prepared by DOC differential solubilization and soluble (sFN) and insoluble (InsFN) fractions of FN were separated by SDS-PAGE. Note the increase in both soluble and insoluble fibronectins in response to Dex treatment. **(C)** 3D spheroids of GBM-3 were generated in the presence or absence of Dex and 50 μg/ml Rhodamine-conjugated FN. Dex treatment resulted in a marked re-organization of FN from punctate to fibrous (left panel). FNMA by spheroids was also assessed by DOC/SDS-PAGE and immunoblot analysis. Note the presence of insoluble fibronectin in response to Dex treatment (right panel).

To further explore whether this variability in FNMA is recapitulated in patient-derived GBM tumor samples, we generated and isolated four primary GBM cell lines. An anti-fibronectin antibody was then used to detect fibronectin fibers by immunofluorescence microscopy. Fig 2A shows that GBM cells assemble FN into either punctate clusters or short fibers and that Dex treatment resulted in the formation of an interconnected meshwork of long fibronectin fibers. This was confirmed by immunoblot analysis. Fig 2B shows that Dex treatment gives rise to a marked increase in the presence of insoluble fibronectin (InsFN), a clear indicator of an increase in capacity for FNMA. In 3D spheroids, Dex also activated FNMA. Confocal sections through 3D spheroids of untreated and Dex-treated GBM-3 show a significant increase in the assembly of fibronectin into fibers in comparison to the more punctate pattern observed in untreated aggregates (Fig 2C, left panel). This was confirmed by immunoblot analysis, which revealed that Dex treatment gave rise to a marked increase in InsFN when compared to untreated controls (Fig 2C). The mechanism by which Dex exerts these effects is likely complicated. Previous studies have shown that Dex activates FNMA in human fibrosarcoma HT-1080 cells by upregulating expression of the diastrophic dysplasia sulfate transporter (DTDST) [30]. Since Dex treatment also activated FNMA in our primary GBM cells, we asked whether it may be working through up-regulation of DTDSD. Fig B in S1 File shows that Dex treatment did not increase DTDSD gene expression in GBM when assessed by qRT-PCR (A). Rather, Dex treatment resulted in a 4-fold up-regulation of α5 integrin gene expression relative to carrier controls as determined by RT-qPCR (B) and by immunoblot analysis (C). We also showed that Dex-treatment increased FN secretion by GBM cells (D). Dex treatment did not appear to increase cadherin expression (E).

### Primary GBM cell aggregates demonstrate tissue liquidity and have variable tissue surface tensions

**Fig 3:**
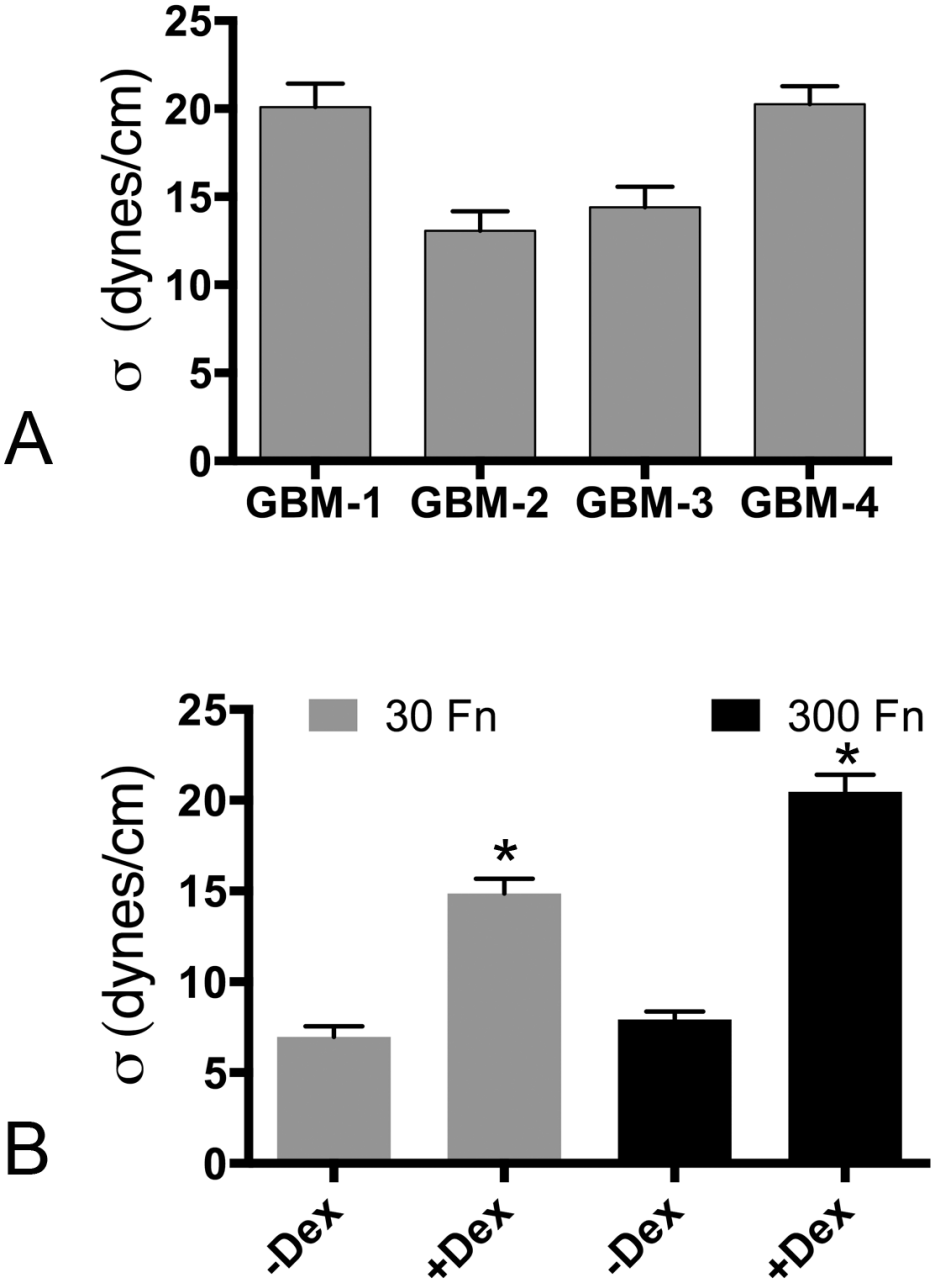
**(A)** The four GBM lines differ in surface tension. ANOVA and Tukey’s MCT revealed that the four GBM lines clustered into 2 groups, with GBM-1 (n = 19) and GBM-4 (n = 20) being more cohesive than GBM-2 (n = 16) and GBM-3 (n = 18). **(B)** Dex treatment significantly increases aggregate surface tension. The low cohesivity line GBM-3 was used to generate aggregates in the absence and presence of Dex and 30 or 300 μg/ml of FN. Dex treatment significantly increased aggregate surface tension in 30 μg/ml FN (n = 22). This increase was more pronounced when aggregates were incubated with 300 μg/ml FN (n = 19). * represents significance for pair-wise comparison by Student’s t-test, p<0.0001.

Our published studies were the first to demonstrate that despite similar histopathological grade, spherical aggregates of 3 commercially available immortalized GBM cell lines differed significantly in their cohesion [31]. Thus we hypothesized that we may also detect differences in aggregate cohesion in primary cultures of GBM. Table 1 provides a comprehensive analysis of GBM aggregate tissue surface tension (σ) for the four primary lines. Spherical aggregates formed from all GBM samples thus exhibiting the defining characteristics of liquid-like behavior: (1) they display a constant calculated surface tension when subjected to two different compressions. Accordingly, the means of σ_1_ and σ_2_ when compared by a paired t-test are not significantly different: (2) the ratio of σ_2_/σ_1_ approaches 1 and is less than the ratio of the applied force at each successive compression (F_2_/F_1_). Table 1 shows that for all lines, a t-test comparing the ratios of σ_2_/σ_1_ to F_2_/F_1_ resulted in a p-value <0.0001, indicating that the ratio of σ_2_/σ_1_ was significantly smaller than that of F_2_/F_1_: 3) the surface tension of the aggregates is independent of aggregate volume. The relationship of aggregate surface tension and volume was analyzed by regression analysis for all lines and the correlation coefficient (R^2^ = 0.085) indicates that sigma is independent of volume (Fig A in S1 File). The TST measurements of the GBM cell aggregates reveal significant differences between the 4 lines (Fig 3A, ANOVA, p<0.0001, Table 1). GBM-1 (20.1 ± 0.9 dynes/cm) and GBM-4 (20.3 ± 1.0 dynes/cm) having significantly higher surface tension than GBM-2 (13.1 ± 1.1 dynes/cm) and GBM-3 (14.1 ± 1.2 dynes/cm). These results demonstrate that despite a common histopathological classification, aggregates of GBM cells have distinct surface tensions, with higher surface tension corresponding to higher cohesivity.

**Table 1:** Tissue surface tension measurements and confirmation of aggregate liquidity for aggregates of primary GBM cells.

Line	σ_1_ (Dynes/cm) ± s.e.m.	σ_2_ (Dynes/cm) ± s.e.m.	σ_1,2_ (Dynes/cm) ± s.e.m.	t-test (σ_1_ vs. σ_2_) p =	σ_2_/σ_1_	F_2_/F_1_	t-test σ_2_/σ_1_ vs F_2_/F_1_ p
GBM-1	21.6 ± 1.9	18.4 ± 1.8	20.1 ± 0.9	0.2488	0.89 ± 0.06	1.43 ± 0.07	<0.0001
GBM-2	13.8 ± 1.6	12.4 ± 1.5	13.1 ± 1.1	0.5355	0.95 ± 0.06	1.50 ± 0.07	<0.0001
GBM-3	14.9 ± 1.4	14.0 ± 2.0	14.4 ± 1.2	0.7231	0.94 ± 0.09	1.45 ± 0.04	=0.0003
GBM-4	21.0 ± 1.5	19.5 ± 1.5	20.3 ± 1.0	0.4839	0.94 ± 0.06	1.57 ± 0.12	=0.0002

### Dex treatment results in increased aggregate compaction and cohesion of GBM aggregates

We have previously shown that activation of FNMA gave rise to an increase in U87-MG aggregate cohesion. We here asked whether this would also be the case for primary GBM cells. We first performed compaction assays in the presence or absence of Dex. For all lines, Dex-treatment resulted in significant aggregate compaction suggesting an increase in intercellular cohesion (Fig C in S1 File). We then selected one of the less cohesive cell lines (GBM-3) to perform TST assays. Previous studies have shown that FN concentration can significantly influence aggregate cohesion [32]. We therefore incubated Dex-treated GBM-3 cells in either 30 μg/ml or 300 μg/ml of rat plasma FN. Fig 3B shows that Dex treatment significantly increased aggregate surface tension in 30 μg/ml FN from 6.7 ± 0.6 dynes/cm to 14.9 ± 0.8 dynes/cm. This increase was more pronounced when aggregates were incubated with 300 μg/ml FN. At this higher fibronectin concentration, surface tension increased from 7.9 ± 0.4 dynes/cm to 20.5 ± 0.9 dynes/cm). It is noteworthy that the TST measurements for GBM-3 in Fig 3A is higher than that of the same line in Fig 3B. This is because in Fig 3A, GBM-3 cells were incubated in TCM with 10% FCS. Fibronectin concentration was undetermined but was likely in the published range of 30–300 μg/ml [33]. Aggregates in panel B, however, were incubated in fibronectin-depleted serum that had been supplemented with a controlled amount of rat plasma FN (30 or 300 μg/ml). Accordingly, aggregates in panel B may have been exposed to lower FN concentrations than of those incubated in TCM/10% FCS. This could account for the lower TST values, at least for the 30 μg/ml Fn group.

### Dex treatment alters cell shape and actin organization

**Fig 4:**
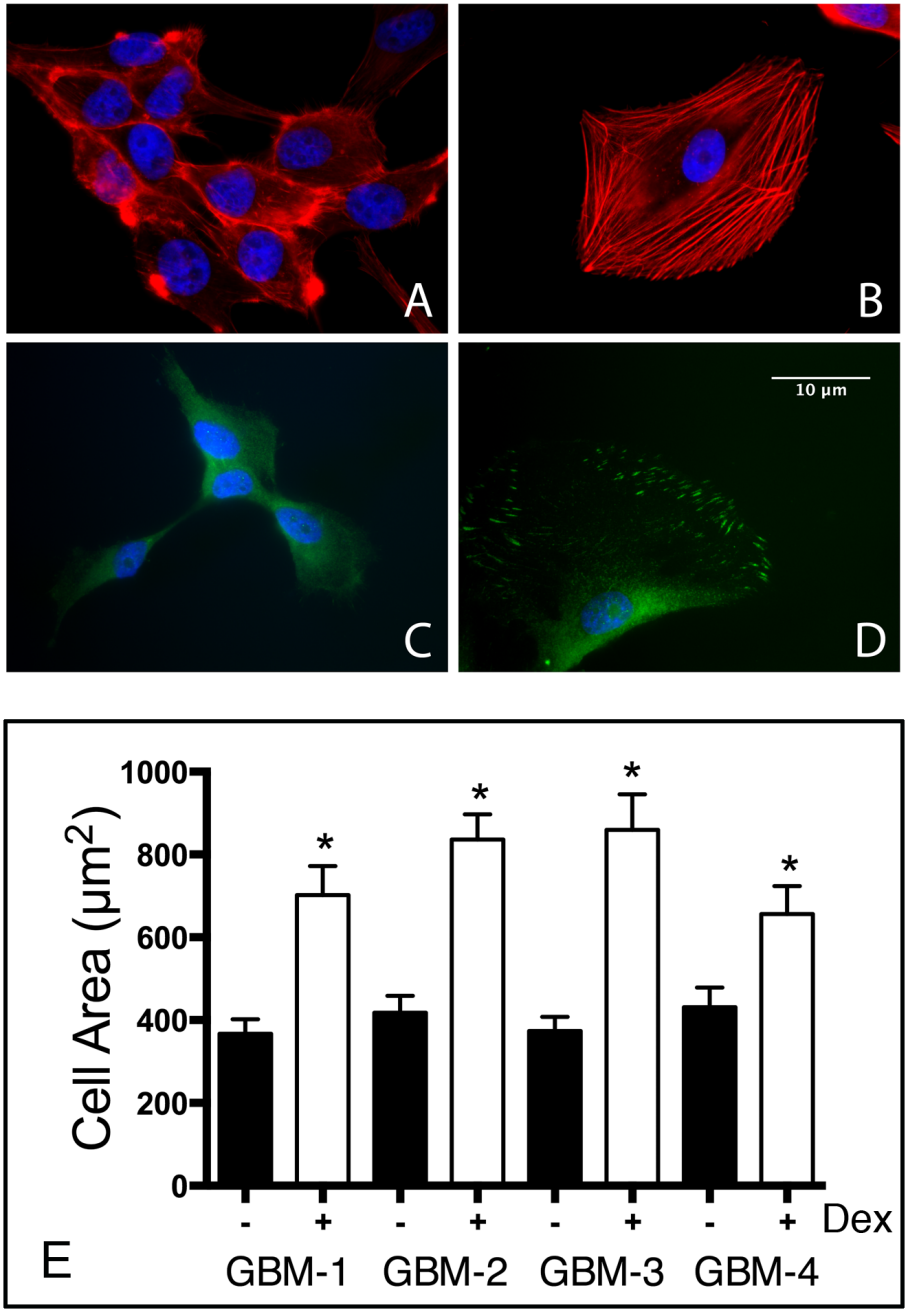
Dex treatment results in a change in cell shape, actin organization and pFAK localization. GBM-3 cells were either left untreated (A, C) or were treated with 10^−7^ M Dex for 24 hours (B, D), whereupon they were fixed and permeabilized and labeled with either rhodamine-phalloidin or with a pFAK antibody, and DAPI as counterstain. Note that Dex treatment resulted in a change in cell shape and a significant reorganization of actin from cortical (A) into stress fibers (B). This was accompanied by a change in the localization of pFAK from cytoplasmic (C) to sites of cell-ECM adhesion (D). ImageJ was used to measure cell area in the absence or presence of Dex for the 4-GBM lines. Pair-wise comparison by Student t-test showed a significant increase in cell size in response to Dex treatment (p<0.05).

Our previous unpublished observations showed that Dex treatment also gave rise to a significant change in U87-MG cell morphology; treated cells adopting a flatter, more adhesive phenotype, and that these changes were associated with a change in actin organization. We therefore asked whether Dex elicited a similar response by primary GBM cells. Fig 4 shows that actin organization in untreated GBM cells is mainly cortical (A). Dex treatment markedly altered the organization of actin into stress fibers. Treated cells also adopted a flatter morphology and appeared to be more tightly attached to the surface (B). The localization of phosphorylated focal adhesion kinase (pFAK) was also altered by Dex treatment. pFAK expression was mainly cytoplasmic and diffuse in untreated cells (C). Upon Dex treatment, pFAK translocated to sites of cell-substratum attachment (D). The translocation of pFAK from the cytoplasm to sites of substrate attachment was also observed in PC3 prostate cancer cells in response to Nestin down-regulation [34]. Overall, Dex-treatment appeared to not only alter cell shape, but seemed to increase cell surface area nearly three-fold, indicating a larger contact area between the cell and the surface to which it adheres (E).

### Dex-treatment results in increased resistance to shear stress induced detachment and reduced cell motility

**Fig 5:**
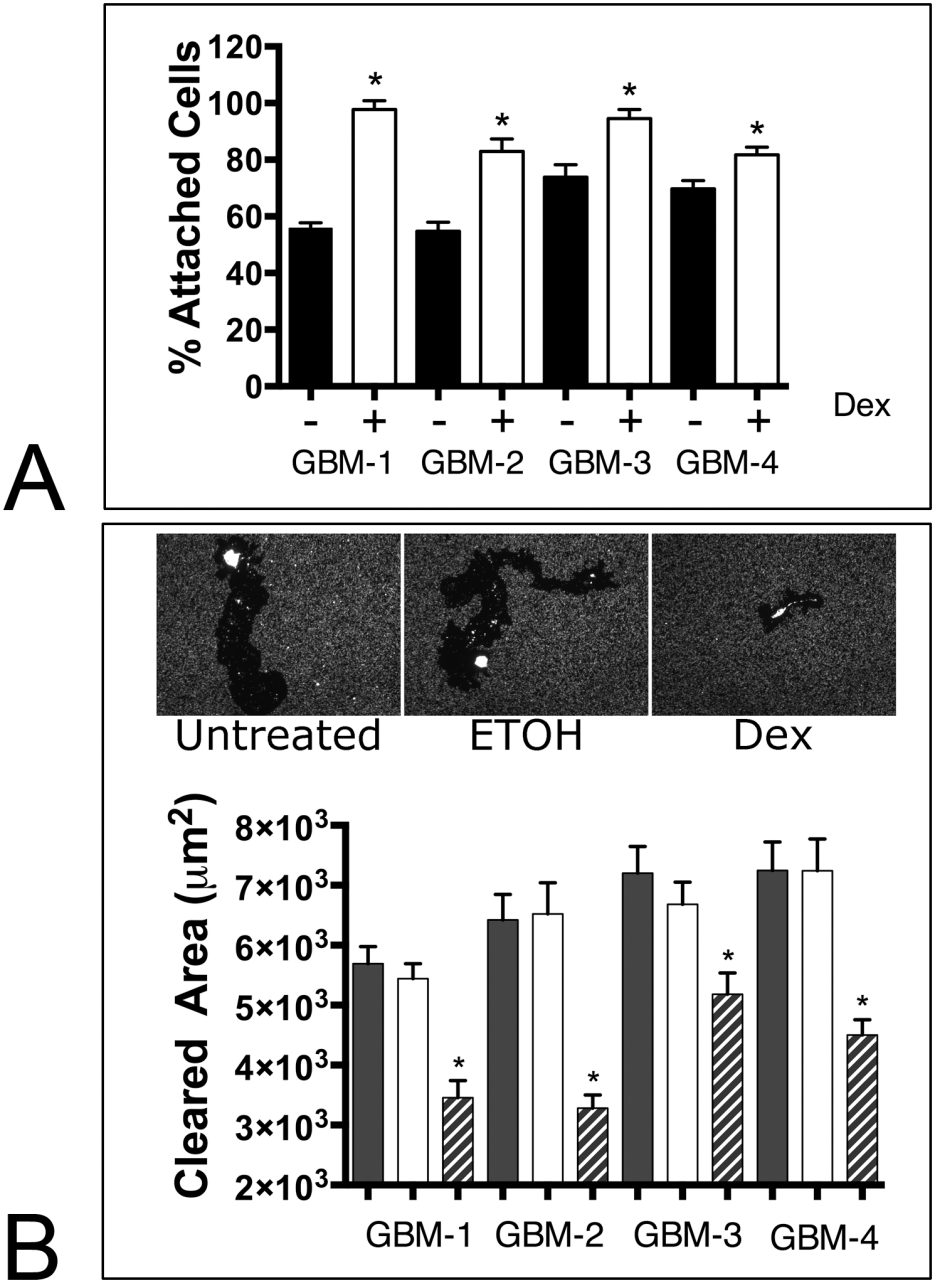
Dex enhances resistance to shear-induced detachment and reduces cell motility. **(A)** Untreated and Dex-treated GBM cells attached to PET membranes were subjected to 30 dynes/cm of shear flow for 3 hours, whereupon the number of cells retained on the membranes was quantified. For all lines, Dex treatment resulted in a significant retention of cells (t-test pairwise comparison, p<0.0001 for GBM-1,2, p = 0.0003 for GBM-3, and p = 0.0042 for GBM-4). Cell motility assays were also conducted in order to determine whether Dex, could also impact cell locomotion. A fluorescent microbead phagokinetic track assay revealed that Dex treated cells were less motile since treated cells appeared to essentially remain in place **(B,** upper panel**)**. Motility was quantified by measuring cleared area of 20 cells for each untreated (solid bars), carrier-treated (clear bars), and Dex treated (hashed bars) groups and by comparing means by ANOVA and Tukey’s MCT. For all GBM lines, Dex significantly decreased cleared area **(B,** lower panel, ANOVA, p<0.0001, asterisk denotes significance, p<0.05**)**.

An increase cell-substrate contact could, in principle, give rise to an increase in the strength of cell-substratum attachment. To assess this, we used a shear-flow assay [35] in which cells were subjected to 30 dynes/cm of flow-induced shear stress for three hours and asked whether Dex treatment rendered cells more resistant to detachment. Fig 5A shows that significant differences in resistance to shear flow exists between the four GBM cell lines (ANOVA, p< 0.0001). Moreover, Dex treatment results in a marked increase in the ability of cells to resist shear stress (ANOVA, p<0.0001). These data suggest that Dex treatment may increase cell attachment strength to substrate. We reasoned that an increase in attachment strength to substrate may hinder cell motility. To assess the effects of Dex on GBM cell motility, we plated dispersed cells onto fluorescence microspheres and measured the non-fluorescent tracks created as cells moved and digested the spheres [27]. In this assay, higher motility corresponds to a larger cleared area. The top panel of Fig 5B demonstrates a representative experiment in which cells were plated onto fluorescent microspheres in either the absence or presence of Dex (with EtOH serving as the vehicle control). Dex-treatment clearly resulted in reduced motility as evidenced by a smaller cleared area. Cell motility was quantified for all GBM cells and cleared area was compared by ANOVA. Fig 5B (lower panel) shows that motility differs between the cell lines (ANOVA, p<0.0001). Moreover, Dex significantly reduced motility in all cell lines (Tukey’s MCT, p<0.05).

### Dex treatment significantly decreases GBM aggregate dispersal velocity

**Fig 6:**
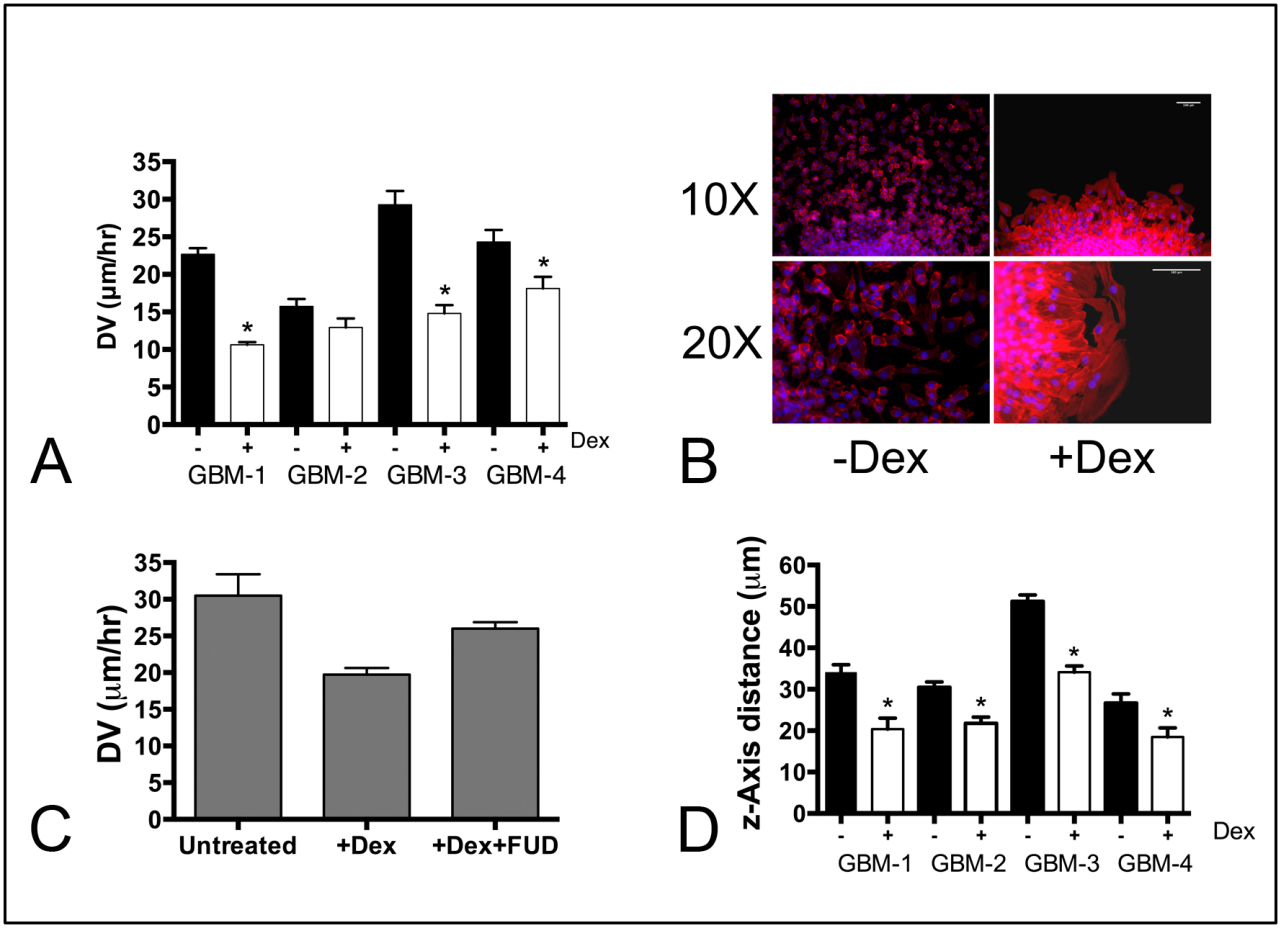
Dex decreases dispersal of GBM cells. (**A**) Aggregates, ranging in size from 50–70 μm in diameter were plated onto tissue culture plastic in complete medium. DV was measured by calculating the change in diameter as a function of time. Regression analysis generated a slope and R^2^ value that were used to calculate velocity. The DV of untreated aggregates (black bars) was significantly higher than that of Dex-treated aggregates (clear bars), for GBM-1,3, and 4. GBM-2 dispersal was reduced, but not significantly (p = 0.12). **(B)** Dex increases cohesion and promotes actin stress fiber formation between cells at the advancing cell front. Note single cell dispersal from untreated aggregates, in contrast to a higher level of cell-cell contact and actin stress fibers in response to Dex treatment. **(C)** One of the high motility lines (GBM-3) was used to demonstrate a functional role for FNMA in reducing DV. Inclusion of the FUD fragment blocked the effects of Dex and restored DV of Dex-treated aggregates to levels similar to those of untreated aggregates (Tukey’s MCT, p>0.05). (**D**) Dex also decreased dispersal of aggregates of GBM cells through a normal human astrocyte seeded 3D scaffold (* represent pair-wise comparison, t-test p<0.05).

Since Dex treatment appears to increase the strength of intercellular cohesion and reduces cell motility, we asked whether this would translate into reduced dispersal velocity. We compared the dispersal velocities for aggregates of GBM cells either in the absence (black bars) or presence of 10^−7^ M Dex (clear bars). Fig 6A shows that spheroids of GBM cells differ in baseline dispersal velocities (ANOVA, p<0.0001), and that Dex treatment reduces DV (pair-wise t-test, p<0.05). For three of the 4-GBM lines, Dex significantly reduced DV of GBM-1, GBM-3, and GBM-4 (pairwise t-test, p<0.05). Dex also appeared to reduce DV in GBM-2, however, the reduction in DV was not statistically significant (p = 0.112). Dex-treatment also resulted in a change in the pattern of dispersal of GBM cells from a spheroidal mass. Whereas, the advancing edge of untreated aggregates dispersed as single cells, the leading edge of Dex-treated aggregates advanced as a sheet. Moreover, actin in advancing cells of untreated aggregates appeared to be cortical, whereas in treated aggregates, actin was arranged in stress fibers (Fig 6B). To confirm a functional role for FNMA, we repeated the DV assay in the presence of the functional upstream domain of Streptococcus pyrogenes F1 adhesin (FUD), a known inhibitor of FNMA [20]. Using GBM-1 as representative cell line, we demonstrated that addition of FUD rescues DV to levels that were significantly greater than those of Dex-treated aggregates (ANOVA, p<0.0047, Tukey’s MCT, p = 0.0012), but not significantly different from those of untreated aggregates (Tukey’s MCT, p>0.05, Fig 6C).

### Dex treatment impedes GBM dispersal through a normal human astrocyte-seeded 3D scaffold

The architecture of the brain into which GBM tumor cells disperse is composed mostly of astrocytes with long cytoplasmic extensions that provide a permeable, three-dimensional network. We established an *ex vivo* assay to better represent the cellular and physical context that GBM cells encounter as they disperse. In this assay, aggregates of GBM cells are plated onto the surface of a porous, 200 μm thick, cross-linked polystyrene 3D scaffold seeded with normal human astrocytes. The NHAs were originally seeded at a concentration sufficiently high to establish a block of astrocyte tissue within the scaffold. In order to disperse, cells must first detach from the aggregate and navigate their way through the astrocyte-seeded scaffold. Fig 6D shows that the four GBM lines are, for the most part, similar in their *ex vivo* dispersal, except for GBM-3 that appears to be significantly more dispersive than the other lines (ANOVA, p<0.0001, Tukey’s MCT). Moreover, Dex treatment appears to significantly reduce the dispersal of GBM cells through astrocyte-seeded scaffolds (pair-wise comparison by t-test, p<0.05). This was likely due to a combination of increased strength of cell-cell cohesion, stronger attachment to substrate, and to decreased motility.

### Dex effects can be blocked by RU-486, a corticosteroid receptor antagonist

**Fig 7:**
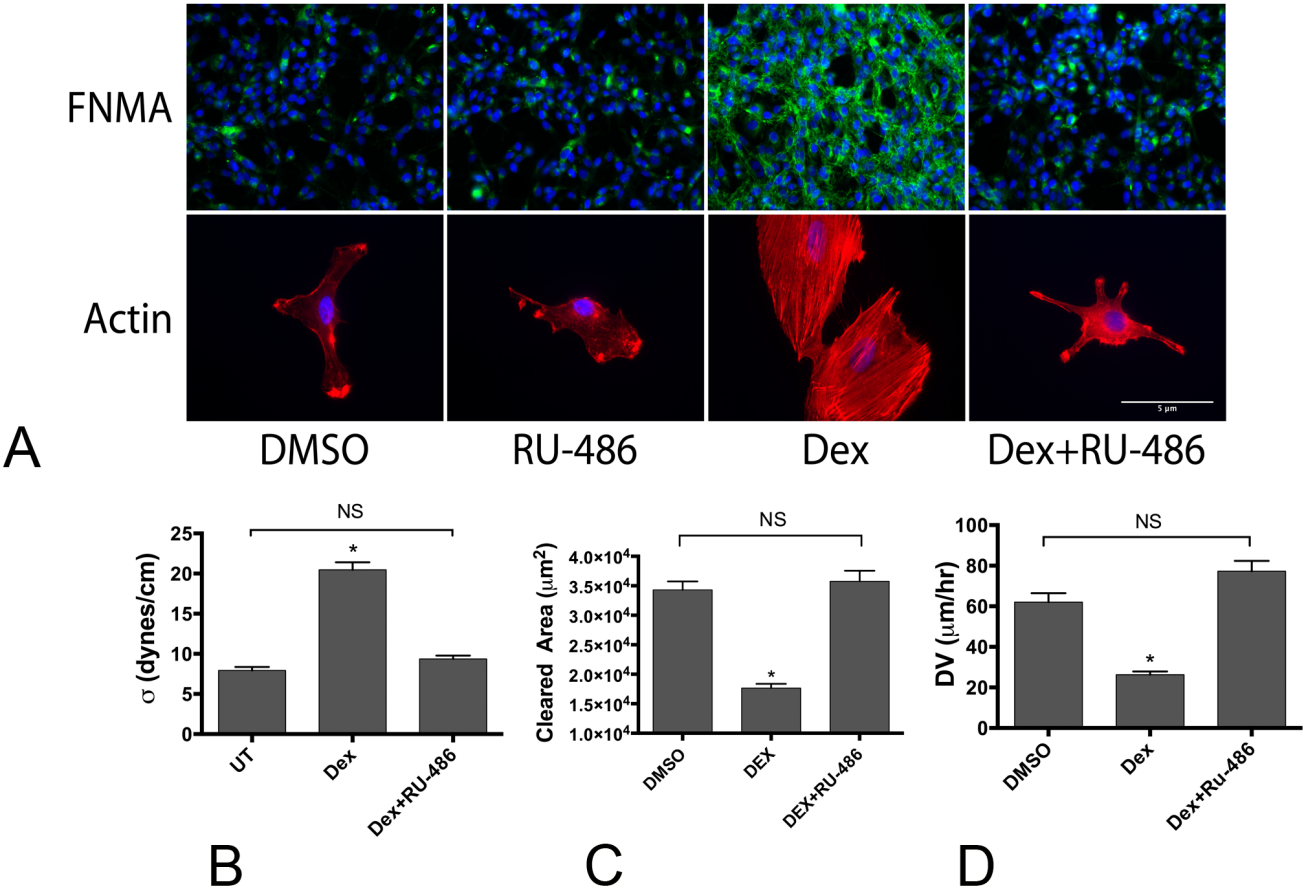
RU-486 blocks the effects of Dex. (**A**) RU-486 blocks Dex-mediated effects on FNMA and actin organization. GBM-3 cells incubated in the presence of Dex and RU-486 fail to assemble a fibronectin matrix and do not reorganize actin into stress fibers. (B). Aggregates incubated in Dex and RU-486 have surface tensions similar to those measured for untreated aggregates. (C) RU-486 appears to block the effects of Dex on cell motility. Combination treatment restores cell motility to levels similar to those of DMSO treated cells. (D) Dex-mediated decrease in aggregate DV can be blocked by RU-486. Dex treatment resulted in a significant decrease in DV compared to DMSO treated aggregates. RU-486 increased DV to levels similar to those of DMSO-treated aggregates (ANOVA, p<0.0001, *** signifies statistical difference by Tukey’s MCT, NS = not significant).

Since Dex works through interaction with corticosteroid receptors, we asked whether the corticosteroid receptor antagonist, RU-486, blocks the observed effects of Dex on GBM. Cells were treated either with Dex alone, or in combination with RU-486. Fig 7A shows that both Dex-mediated increase in capacity for FNMA and actin stress fiber formation were blocked by RU-486. Since RU-486 blocked Dex-mediated increases in FNMA, we asked whether this could have an attendant effect on aggregate cohesion. We measured cohesion by TST of untreated, Dex-treated, or Dex and RU-486 treated aggregates. When aggregates were generated in the presence of both Dex and RU-486, cohesion was markedly reduced and was similar to that of untreated aggregates (Fig 7B). Given the inverse relationship between cohesion and dispersal velocity, we also assessed whether RU-486 would revert cells to a more motile phenotype, and aggregates more dispersive. Fig 7C shows that inclusion of RU-486 reverted Dex-treated GBM cells to a more motile phenotype. This was perhaps associated with the effects of RU-486 on reducing the number of focal adhesion contacts with the substrate (Fig D in S1 File). Collectively, the observation that antagonizing corticosteroid receptors can block the effects of Dex on cell-cell cohesion and on motility, suggests that RU-486 should also block the effects of Dex on DV. Fig 7D shows that whereas Dex significantly reduces DV, inclusion of RU-486 restores DV to that of control aggregates.

### Dex treatment significantly decreases growth rate of 3D aggregates

**Fig 8:**
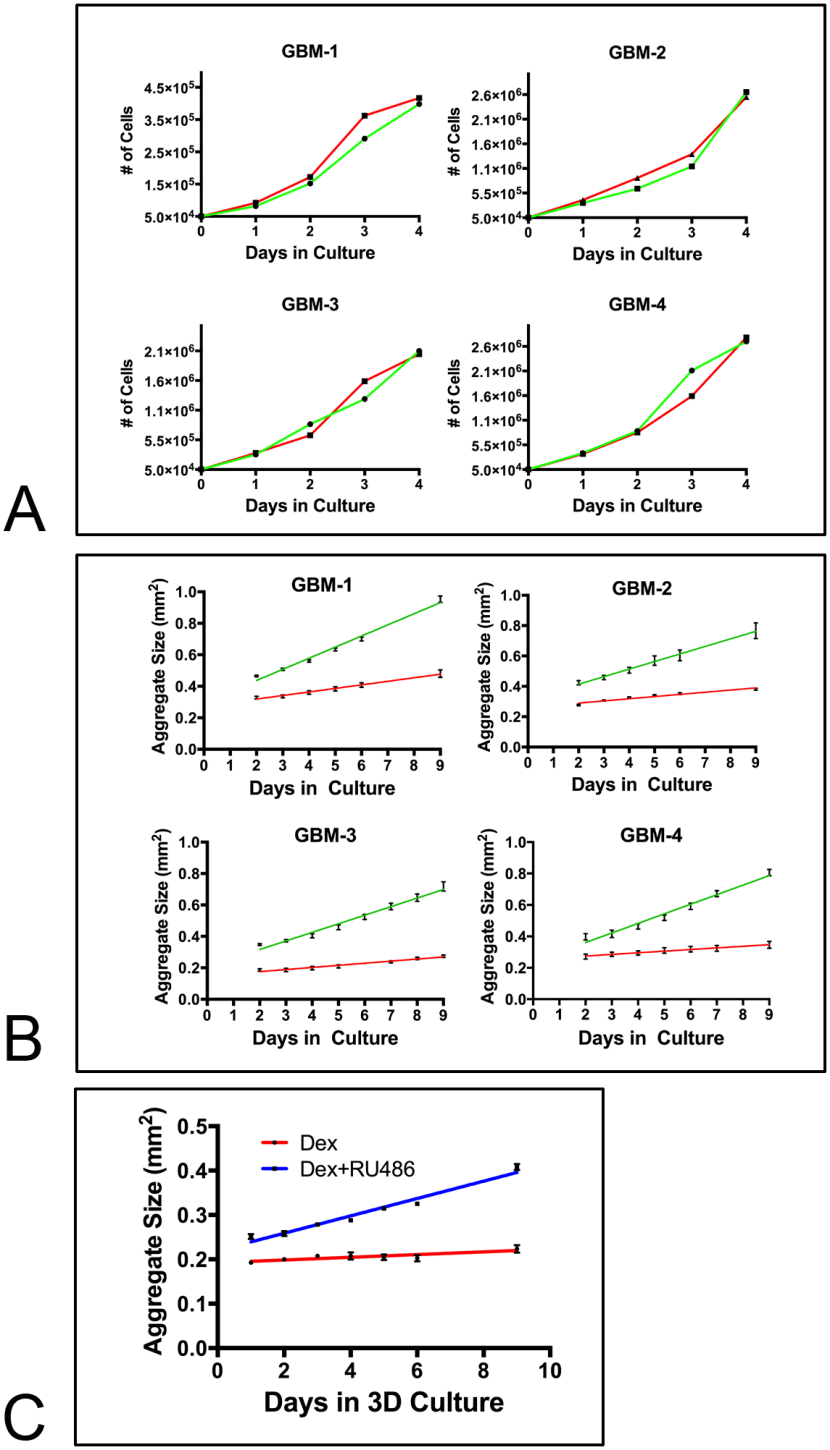
Dex treatment significantly reduces the growth rate of GBM spheroids. **(A)** Untreated (green line) or Dex-treated (red line) GBM were plated as single cells and proliferation was monitored over a 4-day period. Under such conditions, Dex did not appear to influence proliferation. **(B)** When untreated (green lines) or Dex-treated (red lines) GBM were cultured as 3D spheroids for 9-days, growth rate was significantly reduced as demonstrated by a significant shallowing of the slope of the line (ANCOVA, p<0.0001). **(C)** RU-486 blocks the effects of Dex on 3D growth. Spheroids of GBM-3 were incubated for 9 days either in the presence of Dex or in a combination of Dex and RU-486. Note the increase in the slope of the line of the Dex and RU-486 treated spheroids (blue line) as compared to aggregates treated with Dex alone (red line). The difference in the slope equates to a 6.4-fold increase in growth rate.

Release from contact inhibition of growth is a well-documented phenomenon in cancer [36, 37]. Various molecular mechanisms have been identified as potential regulators of this process (reviewed in [38]). Given that Dex treatment appears to increase cohesion of aggregates of GBM cells by activating FNMA, we reasoned that this may have an affect on growth rate. We first tested growth rate in sub-confluent conventional 2D cultures of GBM cells either in the absence or presence of Dex. Fig 8A shows that Dex did not significantly decrease growth rate as compared to controls. We then repeated the experiment using spheroids of GBM cells. Fig 8B shows that Dex treatment significantly reduced the growth rate of GBM aggregates since the slope of the growth curve was significantly reduced by Dex treatment relative to that of control cultures. Linear regression analysis of the 2D and 3D growth curves revealed that for 2D cultures, the slope of the growth curves were not significantly different when cells were treated with Dex (ANCOVA, p>0.05, Table 2). In contrast, the slopes of the curves were significantly different when cells were grown as 3D spheroids (p<0.0001, Table 3). On average, there was a 4-fold reduction in growth rate when spheroids of GBM cells were treated with Dex (Table 3). Incubation of aggregates of GBM in a combination of Dex and RU-486 resulted in faster growth than aggregates incubated in Dex alone (Fig 8C). Collectively, these results suggest that an association exists between tumor cohesion and growth rate in GBM, and that it may be possible to significantly reduce growth rate by increasing the strength of cell-cell cohesion between tumor cells.

**Table 2:** Statistical analysis of growth rates for untreated and Dex-treated GBM cells in 2D culture.

	GBM-1-Dex	GBM-1 +Dex	GBM-2-Dex	GBM-2 +Dex	GBM-3-Dex	GBM-3+Dex	GBM-4-Dex	GBM-4 +Dex
Are slopes non-zero?	P<0.0001	P<0.0001	P = 0.0256	P = 0.0070	P = 0.0027	P = 0.0044	P = 0.0045	P = 0.0077
Are slopes non-linear?	P = 0.5000	P = 0.9000	P = 0.500	P = 0.500	P = 0.500	P = 0.500	P = 0.500	P = 0.500
Are slopes different?	P = 0.1196		P = 0.9952		P = 0.853		P = 0.8083	
Slope	90550	100400	584100	583050	493700	510100	687800	653500
Ratio	0.90		1.00		0.97		1.05	

**Table 3:** Statistical analysis of growth rates of untreated and Dex-treated GBM cells in 3D culture.

	GBM-1-Dex	GBM-1+Dex	GBM-2-Dex	GBM-2+Dex	GBM-3-Dex	GBM-3+Dex	GBM-4-Dex	GBM-4+Dex
Are slopes non-zero?	P<0.0001	P<0.0001	P<0.0001	P<0.0001	P<0.0001	P<0.0001	P<0.0001	P = 0.0006
Are slopes non-linear?	P = 0.3	P = 0.4	P = 0.9	P = 0.4	P = 0.1429	P = 0.1429	P = 0.2	P = 0.8
Are slopes different?	P<0.0001		P<0.0001		P<0.0001		P<0.0001	
Slope	0.0706	0.02253	0.04978	0.01428	0.05457	0.01338	0.0609	0.01035
Ratio	3.1		3.5		4.1		5.9	

## Discussion

This study presents evidence that fibronectin expression and processing varies across GBM tumor samples and that activating FNMA can potentially reduce dispersal and growth of primary GBM cells. We detected 4 distinct patterns of FN expression and organization, ranging from a well-organized matrix to a more punctate pattern, little to no expression or organization, and interestingly, to high levels of expression without secretion or organization. All in all, of the 36 serial GBM samples, only 5 demonstrated normal FN expression and organization, with the remaining 90% of the samples displaying some abnormality in FN expression and organization. These studies are in agreement with the large-scale genomic [28] and proteomic [29] studies which have revealed intratumoral heterogeneity in GBM, and with a more recent analysis of FN expression in these different GBM subcategories [39]. The different patterns of fibronectin expression, secretion, and organization observed in this study suggest that integrin function and other mechanisms regulating fibronectin processing may also be involved.

As mentioned in the introduction, many of the studies connecting integrins to glioma migration have been conducted in immortalized cell lines, many of which were developed nearly 50 years ago. Recent studies, however, have demonstrated that some of the more commonly used cell lines, U87-MG for example, migrate and disperse in vitro, but fail to do so when implanted into organotypic rat corticostriatal slice cultures [40]. Our earlier studies exploring the role of Dex on activation of FNMA and attendant effects on dispersal were informative, but were restricted to cell lines that may have responded differently to Dex treatment than freshly-excised primary tumor tissue. Therefore, we generated four primary cell lines from freshly excised tumor samples and asked whether these lines also showed differences in FN expression and organization. We specifically selected adherent cells for this study, since they comprise the bulk of the tumor and are likely responsible for the mass effect associated with brain tumor-related edema. [40]

We assessed each cell line’s ability for FNMA. Immunofluorescence and immunoblot assays clearly demonstrated a limited but variable capacity for FNMA by these primary lines. This variability has also been observed in existing commercial cell lines. U87-MG cells, for example, can barely assemble a matrix, whereas LN-229 and U118-MG have higher capacity for FNMA [4]. This variability in fibronectin expression and processing by immortalized and primary cells is in line with the variability observed in the GBM microarray. An interesting exception is the pattern observed in Cluster 3 of the GBM array. Here, fibronectin is highly expressed but does not appear to be secreted or assembled into a matrix. GBM-1 cells appear to also have low capacity for fibronectin secretion and matrix assembly. Collectively, the data point to a deficiency in fibronectin expression and processing by GBM tumors.

We also demonstrated that Dex treatment can activate FNMA and that this gives rise, in part, to increased cohesion and decreased dispersal. Our previous study using immortalized GBM cells established a direct relationship between levels of fibronectin matrix and aggregate cohesion. Those studies used 3 commercially-available cell lines that were essentially chosen because they had different capacity for FNMA, very little (U-87MG), moderate levels (LN-229), and significant (U-118MG). As it happens, FNMA and cohesion were directly correlated. This does not appear to be the case for these primary GBM cells. This is perhaps because the primary lines may be significantly different in their expression of various adhesion molecules that could significantly influence their cohesion. What is consistent between the immortalized and primary GBM cells is that Dex treatment ultimately increases FNMA and aggregate cohesion.

All in all, it appears that Dex treatment provides more α5 integrin and FN and this could explain the observed increase in FNMA. We therefore explored whether simply overexpressing α5 integrin in GBM cells could give rise to increased FNMA. This proved not to be the case since transfected cells failed to assemble a FN matrix to any greater extent than untransfected controls (data not shown). This suggests that Dex may also be involved in activating α5 integrin function. The precise mechanism involved, however, is as yet to be elucidated. However, we provide evidence that Dex exerts its effects through interaction with the glucocorticoid receptor (GR). Since the GR requires both agonist binding and phosphorylation to translocate to the nucleus, we used the GR antagonist, RU-486, to confirm that RU-486, significantly reduced capacity for FNMA.

Previous studies utilizing aggregates composed of cells genetically engineered to express cadherins and whose cohesion were specifically tuned, showed that cohesion is inversely proportional to dispersal [5]. This study shows that for GBM, the observed reduction of dispersal velocity in response to Dex treatment was likely due to a combination of increased cohesion that discouraged detachment of tumor cells from the mass, and a decrease in cell motility. Collectively, the redistribution of actin into stress fibers, the change in cell shape to a flatter phenotype, and the increase in pFAK at sites of cell-substratum attachment, stabilized cell-substrate adhesion to a point that significantly restrained cell movement [41, 42]. Collectively, the increase in FNMA, attendant increase in aggregate cohesion, increased attachment to substrate, and decreased motility resulted in a significant decrease in aggregate dispersal velocity.

Of note, is that the pattern of dispersal was also affected by Dex treatment. Whereas, the advancing edge of untreated aggregates dispersed as single cells, the leading edge of Dex-treated aggregates advanced as a sheet. Cells at the advancing front were tightly adherent to one another, suggesting that the Dex-mediated decrease in DV arose as a consequence of increased cell-cell cohesion. Interestingly, similar results were obtained in other studies, where aggregates comprised of FN-deficient cells were able to invade a Matrigel filter, but also demonstrated detachment of cells at the advancing front [39]. These patterns of dispersal have been elsewhere described as a shift from a gaseous (low intermolecular forces) to a liquid (high intermolecular forces) state [43]. The involvement of FNMA in this process was confirmed by incubating Dex-treated cells with the FUD fragment. This fragment specifically prevents the incorporation of soluble FN into a dense matrix. This effectively blocked FNMA and rescued DV, suggesting that the FN matrix was at least partially responsible for the Dex-mediated increase in cohesion and attendant decrease in DV. The DV assays performed here did not take into account changes that could influence DV by modulating strength of adhesion to substrate. However, previous studies have shown that strong-cell-cell cohesion can override strong cell-ECM adhesion, and prevent dispersal [5].

In this study, we demonstrated that activating FNMA in primary GBM cells gave rise to a significant increase in the strength of cell-cell cohesion and reduced capacity for dispersal. We utilized a pharmacologic approach in gain-of-function assays in primary cells that were essentially FNMA deficient. Other studies employed loss of function assays using targeted short-hairpin RNA to deplete fibronectin in U87-MG cells [39]. Those studies concluded that disrupting the fibronectin matrix enhanced persistent directional migration of single cells and compromised collective invasion of spheroids through a laminin-rich matrix. This is consistent with our results. However, as stated previously, glioma cell migration varies widely between cell lines and is highly dependent on the composition of the ECM. Accordingly, care must be exercised when drawing general conclusions from studies using a single cell line. Also, in our experience, U87-MG cells have a very low capacity for FNMA and can only be induced to assemble a matrix if groups of cells are subjected to high tensile forces, such as spheroids that are allowed to spread on a rigid matrix, or allowed to cluster near edges of tissue culture plates. Spheroids of U87-MG grown in hanging drop culture and that are not under tensile stress or that are grown in conventional 2D culture do not typically assemble a matrix.

To disperse, glioma cells must also be able to physically squeeze through pores created by astrocytes that are of smaller diameter than that of their nucleus. To do so, they must undergo shape change and nuclear deformation—processes that require dramatic changes in cytoskeletal organization and in cellular mechanics [44, 45]. We asked whether Dex treatment could also impede the dispersal of GBM cells through an astrocyte-seeded scaffold. Here too, single cell dispersal was significantly impeded by Dex (Fig 6D). Whether Dex increased the stiffness of GBM cells to a point that discouraged their dispersal is currently being assessed.

As discussed above, inclusion of RU-486 abolished Dex-mediated reactivation of FNMA and actin reorganization. We therefore asked whether RU-486 would block the effects of Dex on aggregate surface tension, cell motility, and DV. Inclusion of RU-486, 1) decreased Dex-mediated cohesion to levels similar to untreated aggregates, 2) increased the motility of Dex-treated GBM cells to levels similar to those treated with DMSO, and 3) blocked the effects of Dex on DV by effectively restoring DV to levels similar to those of carrier control treated aggregates. These results indicate that Dex, although having pleiotropic effects, specifically works through the GR pathway to suppress dispersal.

Continued tumor growth after initial resection further complicates treatment efficacy for GBM. Release from contact inhibition of growth is a well-documented phenomenon in cancer [36, 37]. Various molecular mechanisms have been identified as potential regulators of this process, reviewed in [38]. One of the more prevalent mechanisms involves loss of tumor cohesion, as a consequence of reduced E-cadherin expression or function [46]. In GBM, N-cadherin predominates and it’s expression is not upregulated by Dex. However, Dex is able to increase cohesion by reactivating FNMA. We reasoned that an FNMA-dependent increase in cohesion could also reduce growth rate of GBM spheroids. To test this, it was first necessary to demonstrate that Dex did not have an effect on cell proliferation when cells were grown as sparsely-plated 2D cultures. We then performed growth assays on 3D spheroids in the absence and presence of Dex, and for one line, a combination of Dex and RU-486. On average, Dex treatment resulted in a 4-fold decrease in growth rate. Since RU-486 effectively reduced aggregate cohesion to levels of untreated aggregates, we reasoned that RU-486 should also block the effects of Dex on growth rate. This proved to be the case since aggregates treated in a combination of Dex and RU-486 grew 6 times faster than those treated by Dex alone. Collectively, these results indicate that Dex not only has the capacity to decrease the detachment and dispersal of GBM tumor cells, but by increasing cohesion, may also function to reduce the growth rate of 3D spheroids, at least in vitro. Interestingly, several studies conducted in the 1980s also observed a reduction in tumor-size after corticosteroid treatment [47, 48]. These studies, however, were not followed up.

A pharmacologic approach using Dex is logical inasmuch as it is an already approved palliative therapy for brain tumors. Despite its efficacy in reducing edema, side-effects of Dex treatment often limit its long-term use. Accordingly, patients are typically weaned off the drug once edema has resolved [49]. We performed a dose-response experiment to determine the lowest possible dose of Dex that can reactivate FNMA in GBM cells. We show that a concentration of 5x10^-8^ M can fully reactivate FNMA and that even a dose as low as 1x10^-8^ M can also be effective (Fig E in S1 File). This represents a 2–10 fold lower dose than currently used to treat brain tumor-related edema. Interestingly, penetrating brain injury, including tumor resection, triggers upregulation of fibronectin at the injury site [50]. Potential sources of fibronectin in CNS injuries include reactive astroglia and ingressing fibroblasts or Schwann cells, but plasma fibronectin is also an important source. A continuous low-dose pharmacologic strategy administered immediately after resection in an environment rich in fibronectin that is assembled into a dense matrix could reduce dispersal and growth of recurrent tumors.

## Conclusions

GBM almost always recurs. How quickly this happens depends on several factors, including propensity for dispersal and continued growth. Preventing or impeding tumor cell dispersal or reducing tumor growth rate could, in principle, delay the onset of recurrence. We described a potential new role for Dex as a suppressor of dispersal and growth. Dex exerts these effects through a combination of increased cell-cell cohesion, which serves to discourage detachment of tumor cells from the primary mass, and a significant reorganization of actin into stress fibers, which serves to increase the strength of cell-ECM adhesion, effectively reducing cell motility. Dex appears to increase not only the expression of α5 integrin, but also the amount of secreted and assembled fibronectin. However, given its effects on actin reorganization, it is also likely that Dex has an effect of integrin function. The observation that Dex significantly reduces the growth rate of 3D tumor-like spheroids is an added benefit. Dex is routinely used to treat brain tumor related edema but is discontinued once edema has resolved. Indeed, the most recent treatment algorithm for GBM recommends that Dex be tapered or discontinued as soon as possible after surgical resection and radio-chemo therapy. This is partly due to side-effects associated with the high doses employed. We showed that in vitro doses 10-fold lower than the lowest dose currently used, was sufficient to activate FNMA. Perhaps a strategy of prolonged low-dose Dex treatment represents a superior alternative to early steroid tapering. By administering agents that effectively increase cell-cell cohesion and impede cell motility at the appropriate time after initial resection, it may be possible to effectively decrease dispersal. If that agent could also reduce growth rate of the recurrence, while not a cure, may significantly delay the time-course of re-operation and/or other therapeutic intervention for recurrent disease.

## Supporting Information

S1 File Aggregate surface tension is independent of size. For the 4-GBM lines, combined aggregate volumes were plotted as a function of surface tension. Figure A shows that regression analysis generated a correlation coefficient (R^2^) of 0.085, and the slope did not significantly deviate from zero, indicating no relationship between the two parameters. Figure B demonstrates Dex-mediated upregulation of α5 integrin expression. (A) RT-qPCR of DTDST gene expression in HT-1080 (positive control) and GBM cells HT-10 human fibrosarcoma cells have been previously demonstrated to significantly upregulate DTDST in response to Dex-treatment [37]. Here we show that expression of DTDST is upregulated 6-fold in HT-1080 cells in response to Dex treatment. In contrast, GBM cells do not appear to respond in a similar manner. (B) RT-qPCR revealed that Dex treatment resulted in a 2 to 5-fold increase in α5 gene expression. This increase was associated with an increase in α5 protein expression (C) and in FN secretion (D), as assessed by immunoblot analysis. A western blot of GBM lysates probed with an anti Pan-cadherin antibody did not indicate an increase in cadherin expression in response to Dex treatment (E). In Figure C, we show that Dex treatment results in compaction of GBM cell sheets in hanging drop culture. Hanging drop cultures of untreated and Dex-treated GBM cells were incubated for 18 hours whereupon aggregate size was determined. Statistical analysis of aggregate size was by pair-wise comparison of untreated and Dex-treated aggregates using Students t-test. * represent significance difference (p<0.01). Figure D **shows that** RU-486 blocks the distribution of actin and pFAK. GBM-3 cells were incubated either in the presence of Dex or in Dex and RU-486. Actin and pFAK distribution was then assessed by immunofluorescence microscopy. Note that Dex-induced actin stress fibers tend to become less organized in the presence of RU-486. Note also reduction in the number of focal adhesions in the presence of RU-486. Figure E demonstrates that low-dose Dex treatment promotes FNMA in GBM cells. Here, GBM-2 cells were treated with Dex in doses ranging from 1x10^-6^ M to 5x10^-9^ M for 24 hours, whereupon Fn matrix was detected by immunofluorescence microscopy. A dose as low as 1x10^-8^ M was sufficient to promote FNMA.
(DOCX)
