# Supplementary figures and images for: Dexamethasone-Mediated Activation of Fibronectin Matrix Assembly Reduces Dispersal of Primary Human Glioblastoma Cells
Source: PLoS One. 2015 Aug 18;10(8):e0135951. doi: 10.1371/journal.pone.0135951 (PMC4540426; doi:10.1371/journal.pone.0135951)

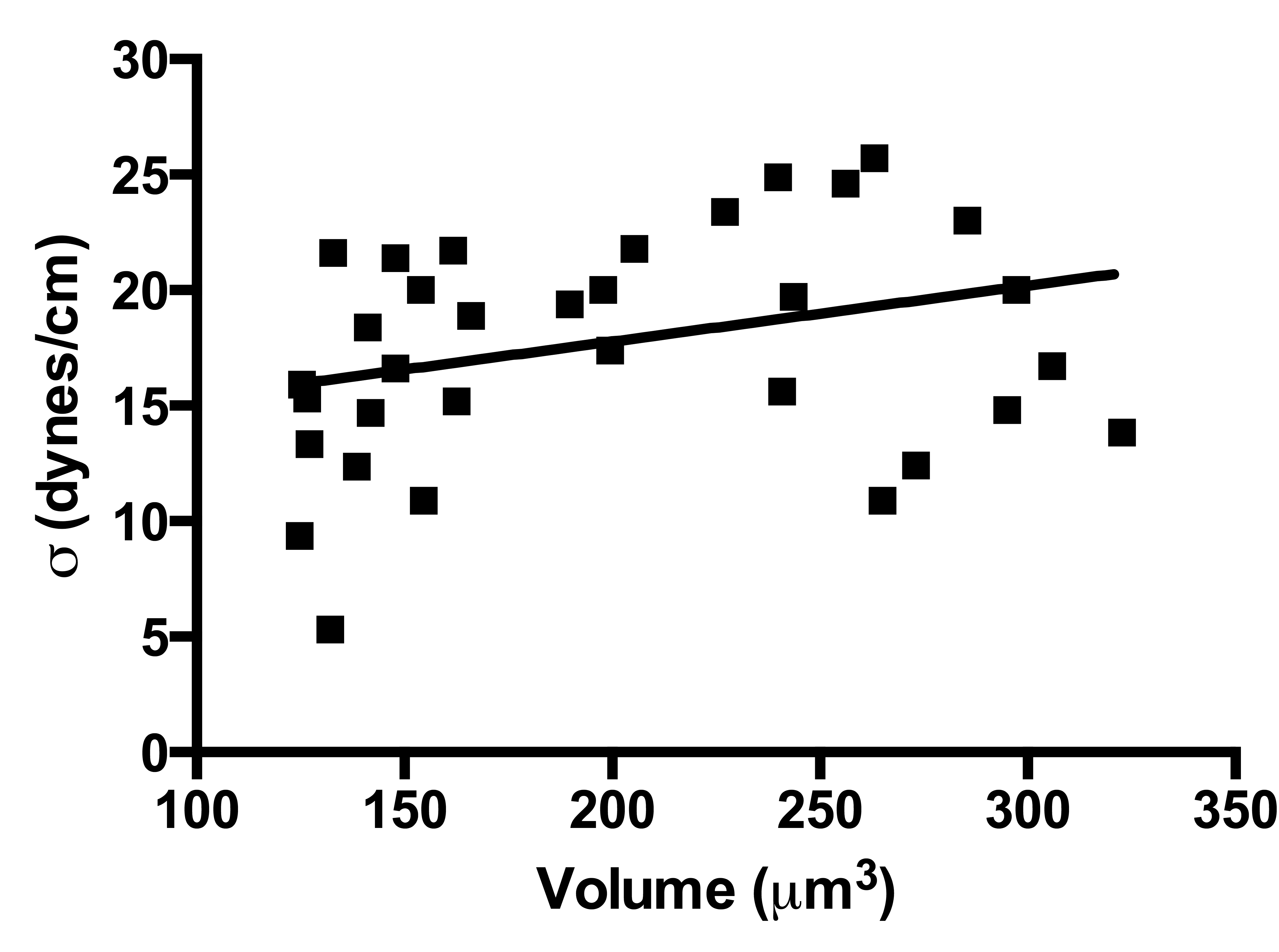
S1 Fig. A

S1 Fig. B


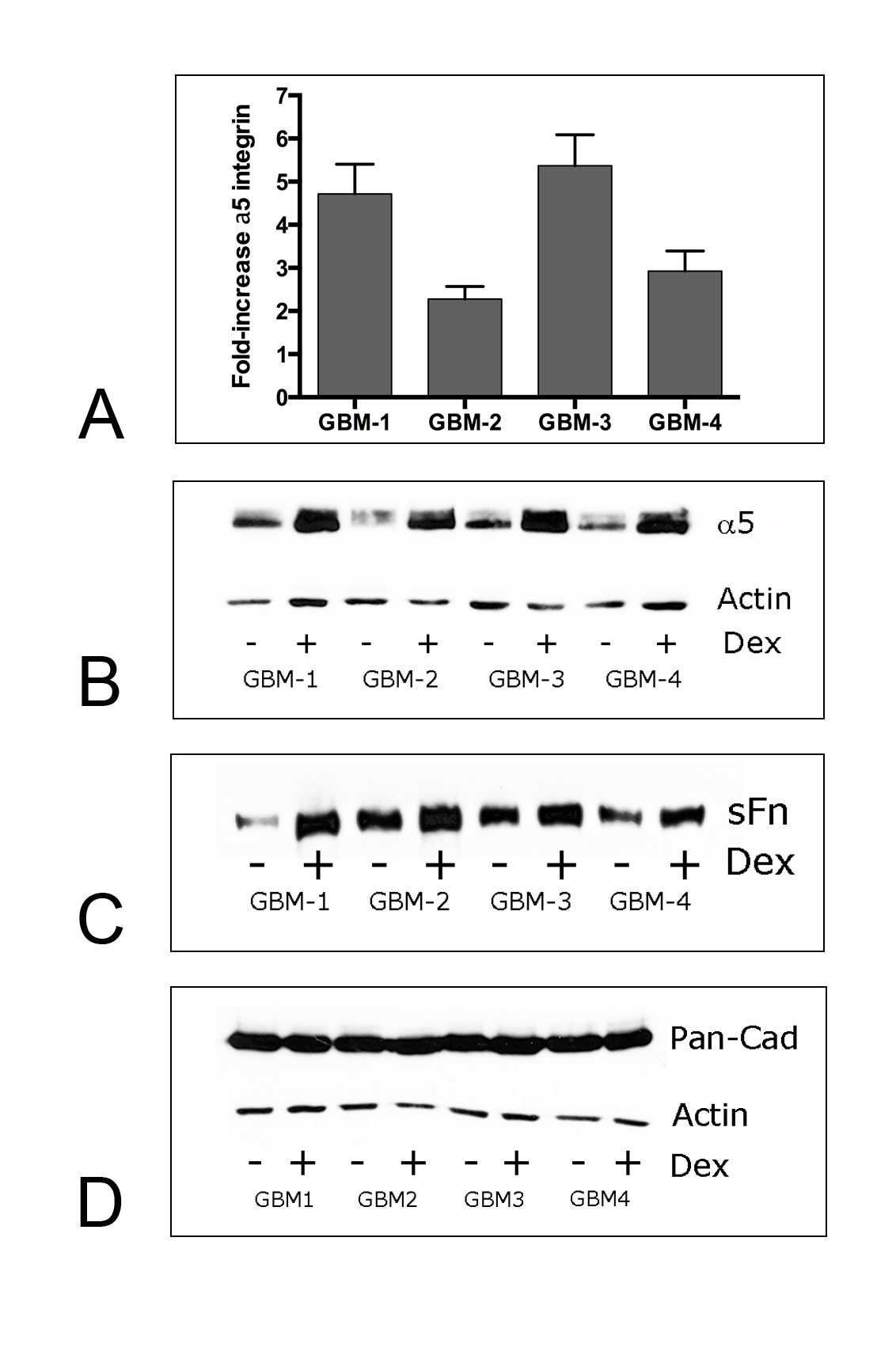


S1 Fig. C


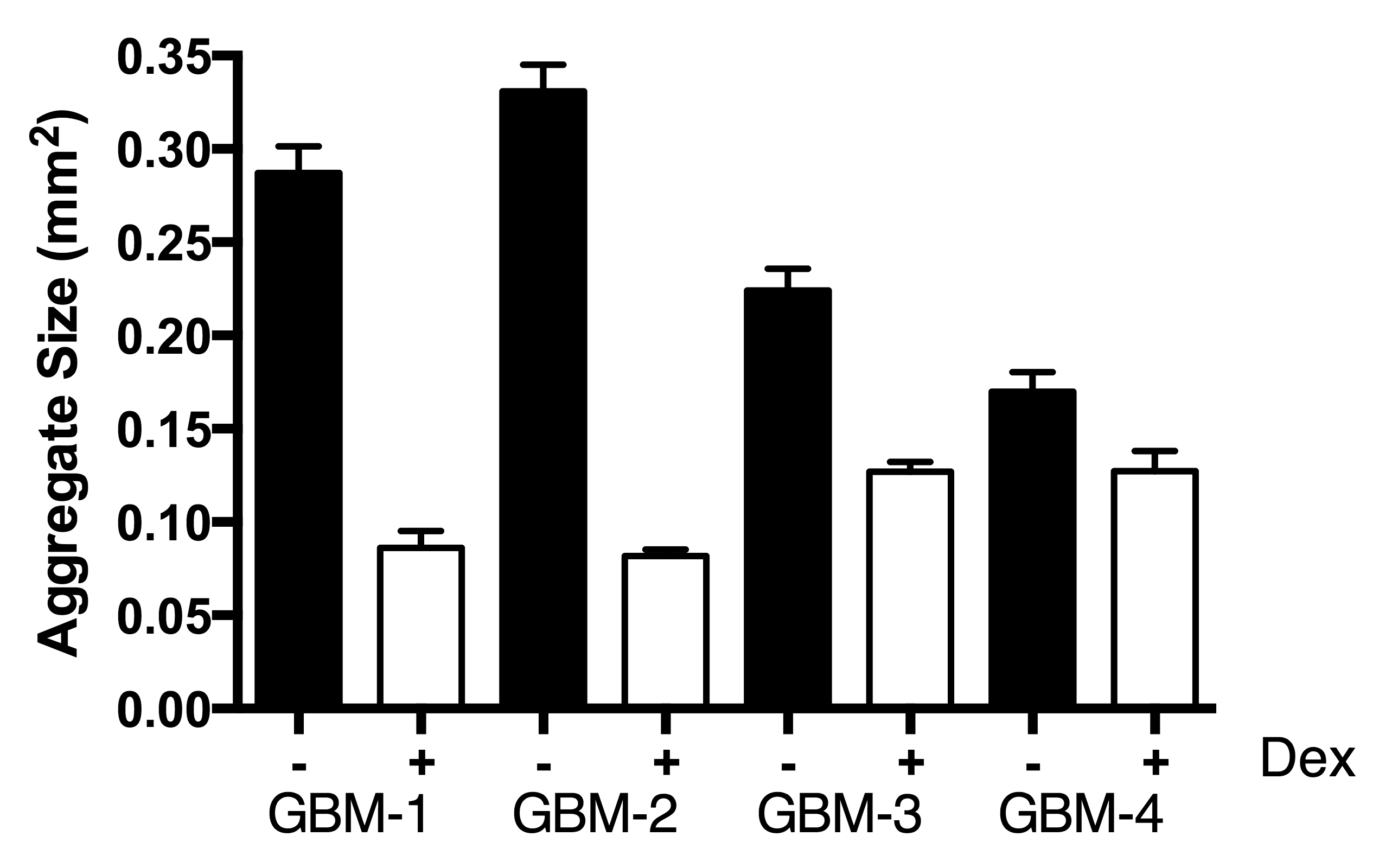


*

*

*

*

S1 Fig. D


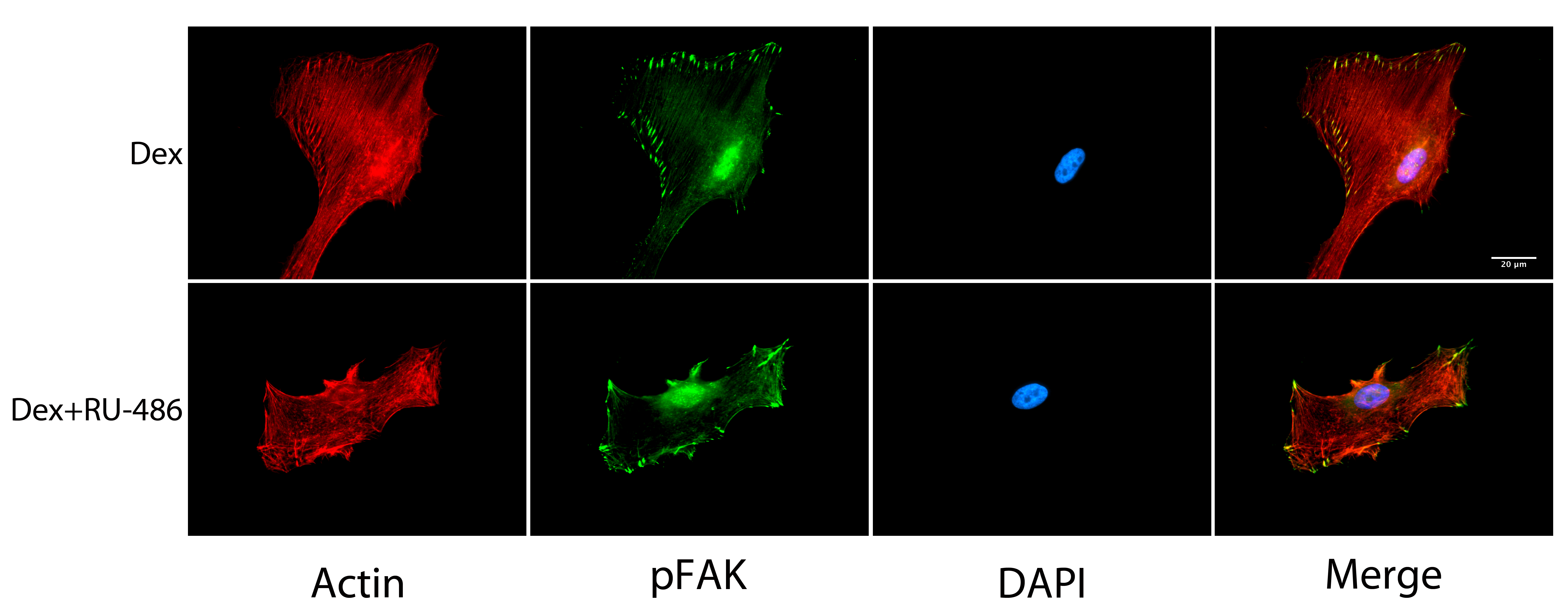


S1 Fig. E


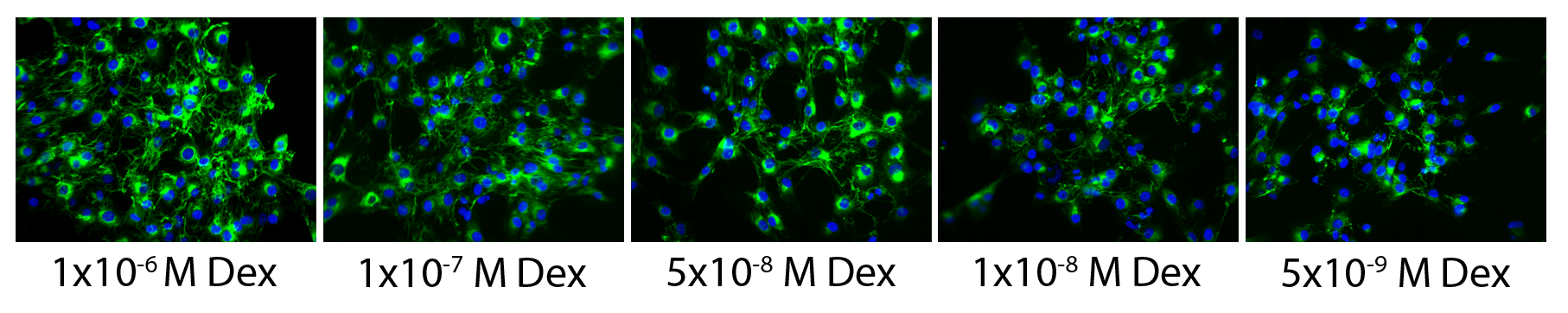

Supplement: S1 File — For the 4-GBM lines, combined aggregate volumes were plotted as a function of surface tension. Figure A shows that regression analysis generated a correlation coefficient (R2) of 0.085, and the slope did not significantly deviate from zero, indicating no relationship between the two parameters. Figure B demonstrates Dex-mediated upregulation of α5 integrin expression. (A) RT-qPCR of DTDST gene expression in HT-1080 (positive control) and GBM cells HT-10 human fibrosarcoma cells have been previously demonstrated to significantly upregulate DTDST in response to Dex-treatment [37]. Here we show that expression of DTDST is upregulated 6-fold in HT-1080 cells in response to Dex treatment. In contrast, GBM cells do not appear to respond in a similar manner. (B) RT-qPCR revealed that Dex treatment resulted in a 2 to 5-fold increase in α5 gene expression. This increase was associated with an increase in α5 protein expression (C) and in FN secretion (D), as assessed by immunoblot analysis. A western blot of GBM lysates probed with an anti Pan-cadherin antibody did not indicate an increase in cadherin expression in response to Dex treatment (E). In Figure C, we show that Dex treatment results in compaction of GBM cell sheets in hanging drop culture. Hanging drop cultures of untreated and Dex-treated GBM cells were incubated for 18 hours whereupon aggregate size was determined. Statistical analysis of aggregate size was by pair-wise comparison of untreated and Dex-treated aggregates using Students t-test. * represent significance difference (p<0.01). Figure D shows that RU-486 blocks the distribution of actin and pFAK. GBM-3 cells were incubated either in the presence of Dex or in Dex and RU-486. Actin and pFAK distribution was then assessed by immunofluorescence microscopy. Note that Dex-induced actin stress fibers tend to become less organized in the presence of RU-486. Note also reduction in the number of focal adhesions in the presence of RU-486. Figure E demonstrates that [file pone.0135951.s001.docx]
